# Discovery of Proteoforms Associated With Alzheimer's Disease Through Quantitative Top-Down Proteomics

**DOI:** 10.1016/j.mcpro.2025.100983

**Published:** 2025-05-05

**Authors:** James M. Fulcher, Ashley N. Ives, Shinya Tasaki, Shane S. Kelly, Sarah M. Williams, Thomas L. Fillmore, Mowei Zhou, Ronald J. Moore, Wei-Jun Qian, Ljiljana Paša-Tolić, Lei Yu, Shahram Oveisgharan, David A. Bennett, Philip L. De Jager, Vladislav A. Petyuk

**Affiliations:** 1 Environmental Molecular Sciences Laboratory, Pacific Northwest National Laboratory, Richland, Washington, USA; 2 Rush Alzheimer's Disease Center, Rush University Medical Center, Chicago, Illinois, USA; 3 Department of Neurological Sciences, Rush University Medical Center; Chicago, Illinois, USA; 4 Biological Sciences Division, Pacific Northwest National Laboratory, Richland, Washington, USA; 5 Department of Chemistry, Zhejiang University, Hangzhou, Zhejiang, China; 6 Center for Translational and Computational Neuroimmunology, Department of Neurology & Taub Institute for Research on Alzheimer's disease and the Aging Brain, Columbia University Medical Center; New York, New York, USA

**Keywords:** top-down proteomics, Alzheimer's disease, amyloid beta

## Abstract

The complex nature of Alzheimer's disease (AD) and its heterogenous clinical presentation has prompted numerous large-scale -*omic* analyses aimed at providing a global understanding of the pathophysiological processes involved. AD involves isoforms, proteolytic products, and posttranslationally modified proteins such as amyloid beta (Aβ) and microtubule-associated protein tau. Top-down proteomics directly measures these species and thus, offers a comprehensive view of pathologically relevant proteoforms that are difficult to analyze using traditional proteomic techniques. Here, we broadly explored associations between proteoforms and clinicopathological traits of AD by deploying a quantitative top-down proteomics approach across frontal cortex of 103 subjects selected from the ROS and MAP cohorts. The approach identified 1213 proteins and 11,782 proteoforms, of which 154 proteoforms had at least one significant association with a clinicopathological phenotype. One important finding included identifying Aβ C-terminal truncation state as the key property for differential association between amyloid plaques and cerebral amyloid angiopathy. Furthermore, various N-terminally truncated forms of Aβ had noticeably stronger association with amyloid plaques and global cognitive function. Additionally, we discovered six VGF neuropeptides that were positively associated with cognitive function independent of pathological burden. The database of brain cortex proteoforms provides a valuable context for functional characterization of the proteins involved in AD and other late-onset brain pathologies.

## Highlights

• Top-down proteomics: 11,782 proteoforms, 1,213 proteins in 103 brain samples.
• C-terminal truncated amyloid beta key for amyloid plaque vs. CAA distinction.
• N-truncated Aβ_x-42_ linked to cognitive decline.
• Six VGF neuropeptides tied to cognitive function, independent of pathology.

## Graphical Abstract

**Figure:**
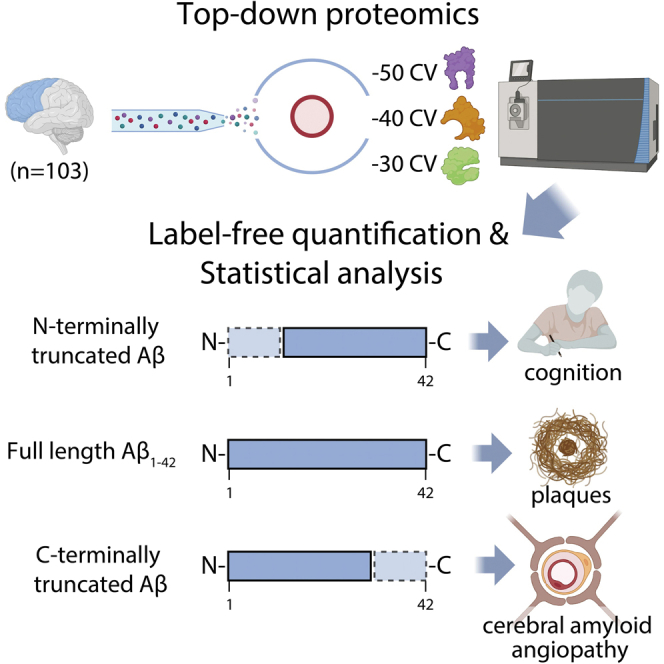

## In Brief

Alzheimer's disease involves diverse proteoforms, including amyloid beta and tau, which are challenging to analyze using traditional proteomic techniques. We applied top-down proteomics to 103 frontal cortex samples from the ROS/MAP cohorts, identifying 11,782 proteoforms. Key findings include amyloid beta C-terminal truncation differentiating amyloid plaques from cerebral amyloid angiopathy and N-terminal truncations correlating with cognitive function. Additionally, six VGF neuropeptides were linked to cognition independent of pathology, providing insights into Alzheimer's disease molecular underpinnings.

Alzheimer's disease (AD) is the most prevalent neurodegenerative disorder, and its incidence has more than doubled over the past three decades in part due to increased lifespan and demographic changes (1). Despite significant investments in dementia research and therapeutic development, a succinct understanding of Alzheimer's remains elusive, and no curative treatments currently exist. Many hypotheses exist on the nature of Alzheimer's etiology, with current leading theories invoking amyloid beta (Aβ), a fragment derived from amyloid precursor protein, and hyperphosphorylation of microtubule-associated protein tau (2). Although these hallmark protein species are well recognized as being components of senile plaques and neurofibrillary tangles, respectively, AD rarely presents with just these neuropathologies. It is commonly observed in combination with TDP-43 inclusions, hippocampal sclerosis, Lewy bodies/Lewy neurites involving α-synuclein protein, and vascular changes including cerebral amyloid angiopathy (CAA) (3). Furthermore, several clinical subtypes of AD have also been described based on neuroimaging and neuropathology studies (4). This complexity has prompted studies that aim to measure large pools of biological molecules in larger sample groups *via* genomics (5), transcriptomics (6), and proteomics (7, 8, 9) to provide new insights into the origins and pathophysiological processes behind AD.

Considering that AD hallmarks involve unusual protein forms, proteomics provides a view that is arguably most directly connected to the underlying pathology. Proteomic techniques most commonly applied require the use of an enzyme such as trypsin to cleave proteins into short peptides that are more amenable for liquid-chromatography mass spectrometric analysis (LC-MS/MS). This is referred to as “bottom-up” proteomics (BUP). Though this approach allows for the quantification of thousands of proteins in a single analysis, the digestion step eliminates the ability to capture the entire molecular composition of the protein. Therefore, the detection of properties such as cleavage by endogenous proteases, single nucleotide variants, post-translational modifications (PTMs), and alternative splicing events are drastically diminished. Collectively, these distinct molecular entities are referred to as “proteoforms” and are best measured utilizing top-down proteomics (TDP) which keeps proteins in their intact primary sequence with PTMs by eschewing enzymatic digestion (10). Because the two hallmarks of AD, Aβ and hyperphosphorylated tau, are noncanonical protein forms, TDP offers many advantages that have yet to be realized in a global TDP analysis of AD. Thus, to broadly explore the association of proteoforms with AD, we deployed a discovery TDP approach to quantify proteoforms across 103 subjects selected from the Religious Orders Study and Rush Memory and Aging Project (ROS/MAP) cohort (11, 12).

**Fig. 1:**
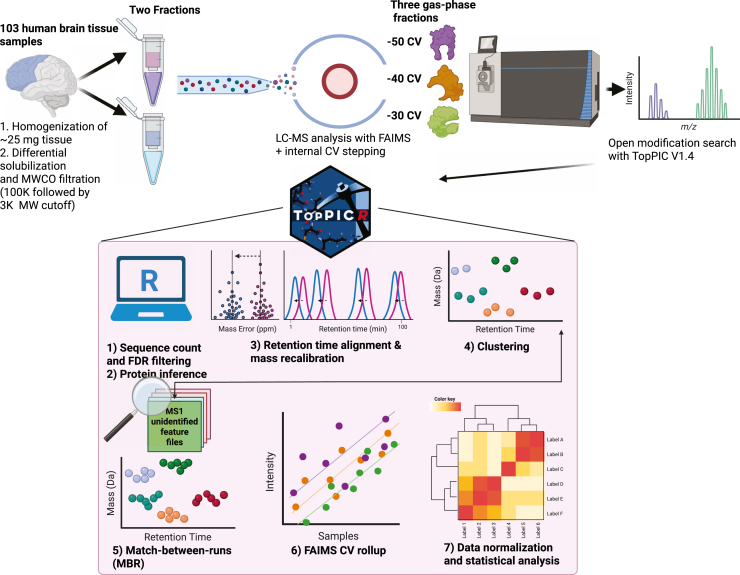
**Workflow for collecting and analyzing top-down proteomics datasets of 103 human brains.** Human brain tissue was processed by differential solubilization, separated into two fractions, and then analyzed by LC-MS with gas-phase fractionation using FAIMS. After database searching with TopPIC, proteoform spectrum match (PrSM) result files are imported into R for downstream analysis with TopPICR. Figure was partially created with BioRender.

TDP, however, is not without its own compromises. It is more limited in proteome coverage compared to the conventional BUP approaches, and tools that enable quantification in a medium/large-scale (>100 samples) TDP studies are at the early inception stage. With regards to low proteome coverage, this can be attributed to several instrument limitations such as rapid ion transient decay in Orbitraps with larger proteins (>30 kDa), signal dilution from having multiple charge states each with isotopologs, and chromatographic peak broadening which leads to overlapping signals during MS acquisition (13). Additionally, Aβ proteoforms are challenging to isolate and analyze due to their inherent propensity for aggregation. Here, we integrate several innovations to mitigate these limitations (Fig. 1). First, the addition of high-field asymmetric waveform ion mobility spectrometry (FAIMS) provides significantly deeper proteome coverage by fractionating ions using compensation voltages (CVs) in the gas-phase based on their relative size and shape (14, 15). Second, we also implemented a new differential solubilization technique utilizing hexafluoroisopropanol (HFIP). HFIP is a potent hydrogen donor that facilitates dissociation of the oligomeric and fibrillar forms of Aβ (16). We found that homogenizing human brain tissue with HFIP could extract Aβ alongside other HFIP-soluble proteoforms into the supernatant, while the pellet could be rehomogenized to provide a fraction with many nonoverlapping proteoforms. Finally, a top-down quantification software (TopPICR (17)) employed several crucial algorithms to reduce data missingness and increase robustness of quantification across the 103 brain samples analyzed. Through the combination of HFIP-differential solubilization, FAIMS, and TopPICR, we demonstrate a novel large-scale TDP approach that enabled identification of 11,782 proteoforms (derived from 1213 proteins) ranging in size from 1 kDa to 28 kDa across 103 subjects and aided one of the deepest TDP studies to date (18, 19).

## Experimental Procedures

### Study Participants

All antemortem cognitive and postmortem data analyzed in this study were gathered as part of the ROS/MAP (11, 20, 21), two longitudinal cohort studies of the elderly within the United States. Both studies were approved by an Institutional Review Board of Rush University Medical Center (ROS IRB# L91020181, MAP IRB# L86121802). Both studies were conducted according to the principles expressed in the Declaration of Helsinki. Written informed consent, an Anatomic Gift Act, and a repository consent was obtained from all ROS/MAP. Clinical and neuropathologic data collection has been previously reported in (22, 23, 24, 25, 26). The entire ROSMAP cohort has also been also extensively characterized with multi-omics approaches (20, 27).

### Mouse Brain Samples

WT C57BL/6 J mouse brains were acquired directly from The Jackson Laboratory. The study was reviewed and approved by the Institutional Animal Care and Use Committee of Battelle, Pacific Northwest Division (#2021-10).

### Experimental Design and Statistical Rationale

For this TDP study, we selected 103 subjects. The rationale behind the subject selection was primarily to ensure that the subjects are well-characterized including genomic, transcriptomic, and epigenomic data. Specifically, we leveraged the same subjects that were used in previous microglia activation studies (28, 29). Selected subjects were representative of the greater study population with respect to age at death and postmortem brain pathology measurements of tangles and Aβ load (Table 1). Specifically, the differences in the following variables were insignificant: age at death (K-S test, *p* = 0.31), AD diagnosis (χ^2^ test, *p* = 0.12), tangles (K-S test, *p* = 0.57), and Aβ load (K-S test, *p* = 0.12). However, there was a significant difference in sex distribution: 72% female in ROSMAP *versus* 61% in the selected 103 subjects (χ^2^ test, *p* = 0.01). This, however, is closer to the general population, where 55% of individuals over 65 years old are female.

**Table 1:** Characteristics and statistics of the 103 participants from the ROSMAP cohort included in this study

Variable	Mean (SD) or n (%)
Demographic	
Age at death (years)	89.4 (5.5)
Female sex (n, %)	63 (61%)
Education (years)	14.9 (2.6)
Postmortem interval (hours)	6.9 (4.6)
*APOE* ε4 allele (n, %)	26 (25%)
Cognition	
Last visit global cognition (score)	−0.83 (1.0)
Slope of cognitive decline (score/year)	−0.015 (0.097)
Postmortem pathological indices	
β-amyloid load (score)	4.7 (4.8)
PHFtau tangle density (score)	6.3 (7.0)
Cerebral amyloid angiopathy (moderate-severe n, %)	36 (35%)
Macroscopic infarcts (n, %)	35 (34%)
Hippocampal sclerosis (n, %)	9 (9%)
Arteriolosclerosis (moderate-severe n, %)	33 (32%)
Neocortical Lewy bodies (n, %)	9 (9%)

Prior to processing, the 103 patient samples were randomized and assigned to six batches (five batches of 18 and one batch of 13 samples). Each sample was analyzed once, resulting in two LC-MS datasets: one for HFIP-soluble and one for urea-soluble fractions. To test the significance of the association between proteoforms and clinical or pathological variables, we used the variables as predictors and modeled proteoforms' log_2_-transformed relative abundances or spectral counts as outcomes. When justified, we included additional clinicopathologic variables or batch effect covariates as predictors. The details of the data analysis itself are provided in the “Preprocessing and Statistical Analysis section” below.

### Sample Preparation

After sample allocation, 15 to 35 mg brain tissue frozen punch biopsies derived from dorsal lateral prefrontal cortex were homogenized on ice with a pellet pestle homogenizer in a 1.5 ml LoBind Eppendorf tube after addition of 8 μl HFIP per 1 mg of wet tissue. Once tissue was fully disrupted, an equivolume amount of homogenization buffer (HB, consisting of 8 M urea, 10 mM ammonium bicarbonate (ABC), 10 mM tris(2-carboxyethyl)phosphine, 2 mM EDTA) was added and then vortexed for 1 min. Samples were incubated at 15 °C for 5 min with 1200 RPM of shaking on a Thermomixer, diluted with HB to a final concentration of 20% HFIP, and then incubated again at 15 °C for 10 min. Insoluble protein was pelleted with centrifugation at 18,000 RCF (10 °C) for 15 min. The HFIP-soluble fraction containing enriched amyloid beta (supernatant) was transferred to a separate 1.5 ml tube on ice. Forty microliters of HB/mg tissue was then added to the pellet before vortexing for 1 min followed by incubation at 15 °C for 5 min at 1200 RPM to extract proteins that were insoluble with the HFIP cosolvent. Both fractions derived from each brain tissue sample were then centrifuged at 18,000 RCF (10 °C) for 15 min before being transferred to separate 4 ml 100 K MWCO spin filters (Amicon) containing wash buffer (consisting of 8 M urea, 10 mM ABC). After each wash step (three total), the flowthrough was transferred to a separate 3 K MWCO filter where it was concentrated/washed three times. After the final wash, the ∼0.1 to 0.2 ml 3 K MWCO filter retentate was transferred to a 1.5 ml LoBind Eppendorf tube where it was acidified to 0.5% formic acid (FA) with a 10% FA stock solution. A bicinchoninic acid assay was then performed using BSA protein standards (0.125–2 mg/ml in 8 M urea, 10 mM ABC, 0.5% FA). Samples were then normalized by dilution with 8 M urea, 10 mM ABC, 0.5% FA to 0.25 mg/ml (HFIP-soluble fraction), or 0.5 mg/ml (HB-soluble fraction) concentration.

### LC-MS Analysis

Samples were analyzed using a Waters NanoACQUITY UPLC system with mobile phases consisting of 0.2% FA in H_2_O (mobile phase A) and 0.2% FA in ACN (mobile phase B). Both trapping-precolumn (200 μm i.d., 5-cm length) and analytical column (100 μm i.d., 50-cm length) were slurry-packed with C2 packing material (5 μm and 3 μm for trap/analytical respectively, 300 Å, Separation Methods Technology). Samples were loaded into a 10 μl loop, corresponding to 2.5 or 5 μg of loaded material and injected onto the trapping column with an isocratic flow of 1% B at 5 μl/min over 15 min for desalting. Separation was performed with a 1% to 50% B gradient over 160 min at 300 nl/min. For MS/MS analysis of proteins, the NanoACQUITY system was coupled to a Thermo Scientific Orbitrap Fusion Lumos Tribrid mass spectrometer equipped with the FAIMS Pro interface. Source parameters included electrospray voltage of 2.2 kV, transfer capillary temperature of 275 °C, and ion funnel RF amplitude of 30%. FAIMS was set to standard resolution without supplementary user-controlled carrier gas flow and a dispersion voltage of −5 kV (equivalent to a dispersion field of −33.3 kV/cm), while the CV switched between three voltages (−50, −40, and −30) throughout data collection (referred to as “internal CV stepping”) (15).

The Fusion Lumos was set to “Intact Protein” application mode, and data was collected as full profile. MS1 and MS2 data were acquired at a resolution of 120k and 60k across a 500-2000 m/z range, with four and two microscans, and AGC targets of 1E6 and 5E5, respectively. MS1 and MS2 were acquired with a maximum inject time of 250 ms. Data-dependent settings included selection of top six most intense ions, exclusion of ions lower than charge state 5+, exclusion of undetermined charge states, and dynamic exclusion after one observation for 30 s. Ions selected for MS2 were isolated over a ± 1.5 m/z window and fragmented through collision-induced dissociation with a collision energy of 35%.

Because of the relatively large sample size of this TDP study, several quality control measures were implemented to ensure the quantitative robustness of the approach. To confirm LC-MS instrument stability, we collected quality control datasets every 3 to 8 samples using an intact protein mixture derived from *Shewanella oneidensis*. Instrument operation was continued only if the number of TopPIC proteoform identifications were above a predefined threshold that was determined when the instrument was known to run optimally. Furthermore, the HFIP differential solubilization sample preparation method was empirically evaluated for its reproducibility. We performed the entire workflow, along with a nonfractionated control, on three biological replicate mouse brain samples. Three technical replicates were collected for each mouse brain sample, and these were spread out over a period of months on the same LC-MS instrument used for our analyses.

### Database Search Parameters and Acceptance Criteria for Proteoform Identifications

Proteoform identification was performed with TopPIC (30) version 1.5.4. Settings for TopPIC included a precursor window of 3 m/z, mass error tolerance of 15 ppm for precursor and fragment ions, a proteoform cluster error tolerance of 0.8 Da, a mass shift upper bound of 4000 Da and lower bound of −150 Da, and a maximum number of allowed unknown modifications of 1. MS2 spectra were searched against the Swiss-Prot database for *Homo sapiens* containing 20,371 reviewed entries, a variably spliced (“VarSplic”) database containing 21,980 splice-isoform entries, and a TrEMBL database containing 57,749 entries (UP000005640- accessed August 29th, 2021). All databases were scrambled to generate decoys which were concatenated during the search. A list of 14 dynamic modifications (N-terminal methionine excision; N-terminal acetylation, acetylation at lysine; C-terminal amidation; N-terminal carbamylation; carbamylation at lysine; deamidation at glutamine or asparagine; methylation or dimethylation at lysine or arginine; iron adduction at aspartic or glutamic acid; phosphorylation at serine, threonine, or tyrosine; N-terminal pyroglutamate; and dioxidation or oxidation at cysteine, methionine, or tryptophan) were provided during the open modification search to reduce the number of unknown mass shifts; no static modifications were specified. The spectra confidence threshold in the TopPIC searches was set at maximum allowable E-value of 0.05.

Downstream data analysis steps were performed in the R environment for statistical computing and TopPICR (17) package. After combining the datasets, we required that a proteoform sequence should be observed in at least two samples, then the E-values were adjusted such that the false discovery rate does not exceed 1% at gene level for each of the three individual types of annotations of the UniProt database: canonical protein sequences (Swiss-Prot), splice isoforms (VarSplic), and tentative protein sequences (TrEMBL). After these filtering steps followed by parsimonious protein accession inference, we achieved the following final false discovery rates: proteoform-to-spectrum match 0.014%, proteoform 0.02%, sequence (proteoform with stripped modifications) 0.21%, UniProt accession 1.7%, and gene level 1.9%. Manual MS/MS validation was performed using LcMsSpectator 1.1.7158.24217 using a precursor and product ion tolerance of 10 ppm. A minimum S/N threshold of 1.5 and Pearson correlation threshold of 0.7 were used for fragment ion assignment.

### Preprocessing and Statistical Analysis

The TopPIC output was converted to MSnSet Bioconductor object using TopPICR package (17) for convenience of the downstream processing and quantitative analysis. Two types of objects were generated: (1) based on MS1 feature intensities and the other (2) using spectral counts as abundance measures. Principal component analysis revealed that processing batch is a significant contributor explaining the variance in the data. This was confirmed by an ANOVA statistical test using the limma package (31). The batch effect was corrected in MS1-based data using the ComBat approach (sva package) (32). Unfortunately, no statistical methods have been developed to correct batch effects in proteomics spectral counting data. Thus, the batch was used as a confounding factor in the statistical modeling of the count data.

As for statistical testing, we used a linear modeling approach for MS1 data (limma package). As for the spectral counting, we used quasi-Poisson–generalized linear modeling approach, known as QuasiTel (33). Besides the sample processing batching factor, we considered other technical (*e.g.* postmortem interval) and demographic (*e.g.* age at death and sex) covariates. Out of all the considered variables, only postmortem interval was statistically significant. Therefore, it was added to the models as a covariate.

Proteoform clustering was performed using the weighted gene correlation network analysis WGCNA tool (34) on MS1 intensity-based data. We required that each proteoform have measurement values in more than 50 samples, which resulted in a final set of 6029 proteoforms. To ensure that clustering was not driven by confounding factors, the effect of postmortem interval was regressed out from the log_2_ ratios of the relative abundances. Missing values were imputed using the svdImpute method from the pcaMethods package (35). Finally, log_2_ ratios were transformed into z-scores by scaling the proteoforms' SDs to ensure that distances reflected the correlation between proteoforms. The WGCNA settings were kept at default, except for the cut-tree distance in the merging step, which was lowered from 0.2 to 0.15.

### Code Availability

The analysis pipeline used in this study and the code reproducing most of the figures is publicly available on GitHub at PNNL-Comp-Mass-Spec/ROSMAP103_TopDown repository.

## Results

### Coverage of the Proteome

**Fig. 2:**
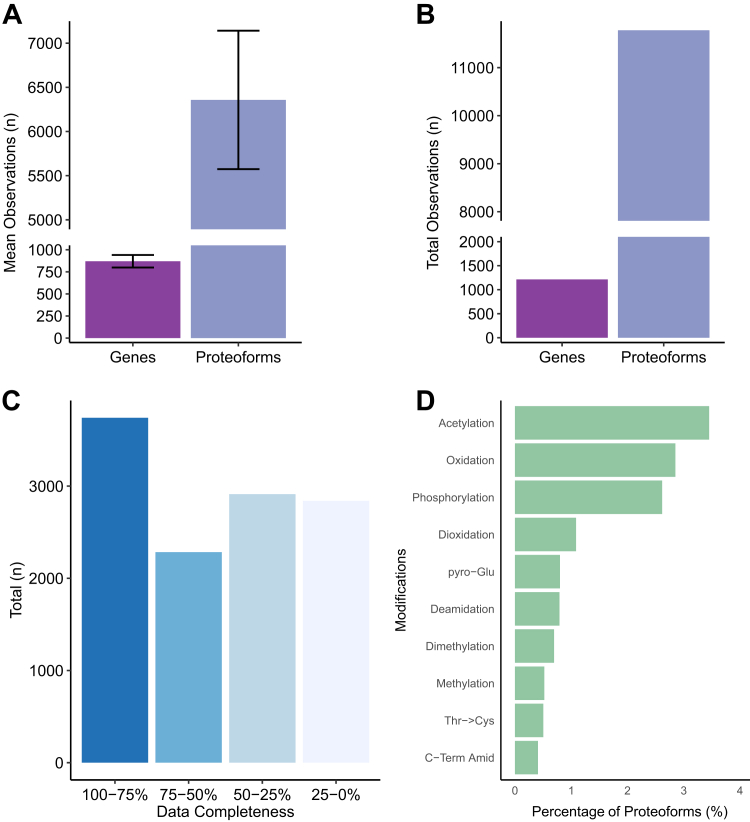
**Overview of the top-down data.***A*, mean number of unique genes and proteoforms observed from each human subject. Error bars represent ± sd. *B*, total unique proteoforms and genes found in the entire study. *C*, percentile bins containing proteoforms with different degrees of data completeness. *D*, top 10 posttranslational modifications found across the entire study, expressed as a percentage of all proteoforms found with the stated modification.

The combination of two HFIP fractions, three FAIMS CVs, followed by LC-MS/MS analysis on average identified 6361 ± 784 proteoforms corresponding to 870 ± 71 genes (Fig. 2*A*) per subject. Combined over 103 samples, we identified 11,782 unique proteoforms corresponding to 1213 genes (Fig. 2*B*).

We applied two approaches for quantifying proteoforms in this study: MS1 intensity-based and spectral count-based. Conceptually, the approaches are similar to their BUP counterparts (36, 37). While the intensity-based approach can be regarded as more accurate and precise, the advantage of the semiquantitative spectral counting approach is that count-based statistical modeling does not require a special treatment of the missing values, since they are treated as zero counts. For the intensity-based data, we required the intensity values to be present in at least 50 samples out of 103 samples. Overall, 6124 proteoforms derived from 836 genes were measured using MS1 intensity-based approach with missing values in fewer than 50% of the samples (Fig. 2*C*) (Supplemental Table S1). In the case of spectral counting, we imposed the requirement that a proteoform must present in at least six samples (3 batches with at least two counts). This resulted in quantifying 7510 proteoforms corresponding to 952 genes (Supplemental Table S2).

The number of identified proteins translates into about 6% of the human proteome (20,361 proteins). However, the current shotgun (*i.e.* nontargeted analysis of an isolated protein) TDP is generally limited to proteins less than 25 kDa (38, 39). Indeed, almost all proteoforms we identified are below 25 kDa (Supplemental Fig. S1). This corresponds to ∼20% of the proteome if measured in its intact form. Notably, the largest full-length protein we could identify in our study was a 28 kDa brain-specific splice variant of tropomyosin (TMBr-3) (40). As proteins are often endogenously cleaved *in vivo,* the proteome coverage also extends to proteins with full-length molecular weights greater than 25 kDa (Supplemental Fig. S1). Although we identify proteoforms derived from proteins far beyond 25 kDa, the bias towards shorter proteins relative to the entire Swiss-Prot database is still present (Supplemental Fig. S1). Proportionally, 89% of our proteoform identifications can be assigned to canonical Swiss-Prot entries, 7% assigned as splice isoforms, and 4% derived from unreviewed TrEMBL entries.

### Efficiency of Aβ Solubilization

Aβ proteoforms involved in plaque formation typically require unique extraction and solubilization procedures. A “gold standard” approach for the plaque solubilization utilizes 70 to 100% FA (41, 42, 43). However, this approach may not be compatible with TDP as it introduces artifactual formylation unless the sample preparation is performed at temperatures ≤ −20 °C (44). Moreover, we desired not to tune the sample preparation method to a specific protein or proteoform in order to capture a greater breadth of proteoforms in our shotgun approach. Therefore, we applied a more generalizable sample preparation method that could also capture Aβ proteoforms efficiently. Recognizing 8 M urea as a well-established chaotropic agent in protein solubilization, we additionally considered HFIP based on prior studies showing its efficiency for solubilization of Aβ fibrils (45). Furthermore, the combination of both has been demonstrated to facilitate solubilization of hydrophobic transmembrane peptides (46). Thus, we deployed a hybrid extraction method that relies on both HFIP and 8M urea to provide two fractions of proteoforms for analysis. The first fraction represents proteoforms soluble in ∼50% HFIP, 8 M urea, while the second fraction represents proteoforms soluble in just 8 M urea. To evaluate the performance of the HFIP/urea method with respect to extraction of the aqueous-insoluble Aβ species, we compared the intensity of our observed Aβ proteoforms to those reported in the prior 5xFAD mouse-based study (47). In this study, the Aβ species were separated into soluble and insoluble by ultracentrifugation followed by denaturation in 1.5 M or 4 M guanidine-based buffer, respectively. The correlation plots comparing intensities of the Aβ species between HFIP method and soluble/insoluble fractions of the 5xFAD mouse-based study are shown on (Supplemental Fig. S2). The Aβ species extracted from the elderly human brains using HFIP method matched best the insoluble species from the aged mice. Thus, the HFIP method is not limited to aqueous-soluble Aβ species. The correlation between Aβ species from aged human brains and insoluble Aβ species from mice increases with mouse age. This supports the use of aged 5xFAD mouse models for studying Aβ pathology.

### Coverage of Protein Modifications

We next investigated the distribution of protein modifications (cotranslational and posttranslational) across all proteoform identifications. Since the modifications were assigned purely based on the matching of the mass to the UniMod database within 0.1 Da tolerance, which is equivalent to 10 ppm for a 10 kDa proteoform, the results should be interpreted with caution. Some modifications can be mistaken for deisotoping errors, such as deamidation. Others may result from artificial modifications introduced during sample preparation, like carbamylation combined with an isotopic error being confused with acetylation. Additionally, some modifications can be misidentified as nonsynonymous mutations, such as an Ala - > Ser mutation being confused with oxidation. Nonetheless, as expected, the most frequently identified modification was N-terminal acetylation (26%), typically introduced during the process of translation. Many proteoforms also contain lysine acetylation (4%), oxidation (3%), phosphorylation (2%), and deamidation (1%) (Fig. 2*D*). The glutaminyl cyclase-mediated N-terminal pyroglutamate (pyro-Glu) (∼1%) is abundant and a known modification present on some neuropeptides and found on several Aβ proteoforms as well (48, 49). We also identified N-terminal myristoylation (∼210 Da), a type of lipidation, on several proteins known to carry this modification including HPCAL1, MARCKS, and VSNL1. We also observed myristoylation on MMP24OS, which has not previously been annotated with this modification (Supplemental Fig. S3).

The largest detected PTMs were glycosylations ranging in size from 161 to 3937 Da that were assigned to previously documented glycoproteins (Supplemental Table S3) (50, 51, 52, 53, 54, 55, 56, 57, 58, 59). These include single hexose modifications of hemoglobin subunits alpha and beta (HBA1, HBB) (53), as well as attachments of more complex sugars to TSC22D1, TSC22D3, HSPE1, CHGA, MAP2, EEF1A1, NDUFAB1, UBB, and NAP1L1 (50, 51, 52, 53, 54, 55, 56, 57, 58, 59). This list of glycan assignments was manually curated to ensure that these large mass shifts were not misidentified based on protein isoform differences, that is, isoforms that differ by the insertion of several amino acids. Additionally, glycosylated proteins undergo preferential cleavage of the glycosidic linkage during MS/MS *via* collision-induced dissociation (60, 61). This is evident by neutral loss of glycosylation from the assigned fragment ions, as well as neutral loss of glycosylation from the parent ion, which we leveraged as evidence of glycosylation (Supplemental Fig. S4). Other unique modifications included uncommon amino acids and single nucleotide variants. For example, incorporation of selenocysteine was readily detected on selenoprotein W (SPEW1). Furthermore, we could distinguish two isomeric haptoglobin allele variants (*HP∗1F* and *HP∗1S*) across different haptoglobin proteoforms. These are differentiated by two missense mutations that lead to an isomeric exchange in amino acids (^129^NE^130^-->^129^DK^130^, Supplemental Fig. S5).

### Association of Protein Cleavages with Unstructured Regions

Among the types of PTMs that can occur, proteolytic cleavage events represented the most frequently observed modification. Over 75% of the proteoforms identified were found to contain at least one cleavage event. Given the frequency of their observation, we sought to identify any patterns or trends that associated with proteolytic cleavage. Because protease cleavages are typically limited to specific features such as primary sequence motifs or secondary structures, we decided to test if protein local structure correlated with cleavage events. We used the AlphaFold2 predicted local distance difference test (pLDDT) score (62), a per-residue estimate of structural confidence, to quantify structure in a given region. We then curated a list of proteoforms where cleavages occurred after the first three amino acids or before the last three amino acids of a given gene sequence (based on the canonical gene sequence). We then averaged the pLDDT scores within ±3 amino acids surrounding a given cleavage site and compared these values to the distribution of pLDDT scores for all amino acids of the detected cleaved proteins (Supplemental Fig. S6). We found a clear shift towards lower pLDDT scores for cleavage sites *versus* the reference data, indicating cleavage sites are enriched in regions predicted to be unstructured or potentially intrinsically disordered (regions with pLDDT <50) (63).

### Reproducibility of the Quantification

#### Repeated Analysis of Mouse Brain Samples

Since label-free top-down proteome quantification is a nascent technique, it is crucial to evaluate its accuracy and reproducibility. To this end, we processed six mouse brain tissue samples using the same HFIP/urea solubilization. This set of resulting 12 fractioned samples was analyzed independently three times in 2 to 3 months intervals. The top-down LC-MS datasets were processed in a similar fashion with TopPIC followed by the companion TopPICR R package. As anticipated, a substantial factor explaining the variance of the data was the batch effect (Supplemental Fig. S7*A*). ComBat procedure (32) effectively removed batch effect resulting in the grouping of repeated LC-MS analyses of the same sample together (Supplemental Fig. S7*B*). The batch correction procedure also significantly reduced the coefficient of variation or relative SD (we avoid using commonly used CV abbreviation to avoid confusion with FAIMS compensation voltage). After the correction, the median coefficient of variation value dropped from 42.3% down to 31.7%, which is on par within batch values (32.1, 31.8, and 31.9%) and similar to values that are often achieved with label-free BUP methods (Supplemental Fig. S7*C*) (64).

#### Correlations with Bottom-Up Datasets

One way of validating accuracy of the data acquired with a new method is to compare it, typically by computing Pearson correlation (R) coefficients, with the measurements acquired using a more established approach. There are two types of prior BUP data that have been collected on the same samples: targeted selected reaction monitoring (65) and shotgun tandem mass tag (8). Having two BUP datasets allows us to establish a reference correlation value of two independent measurements of the same protein. The overlap between the selected reaction monitoring and tandem mass tag datasets encompassed 400 samples and 219 proteins. The median correlation value (R) between these bottom-up measurements is 0.2 (Supplemental Fig. S8). However, the correlation is stronger if a protein has a substantial variance in abundance across the samples, though these abundance fluctuations may not necessarily be relevant to AD or related phenomena. The strongest correlating protein is APP (R = 0.8), peptides of which capture the variation of Aβ abundance. Other high-correlation genes include C1QA, VGF, C1QC, and GPNMB with R values ranging from 0.68 to 0.71.

**Fig. 3:**
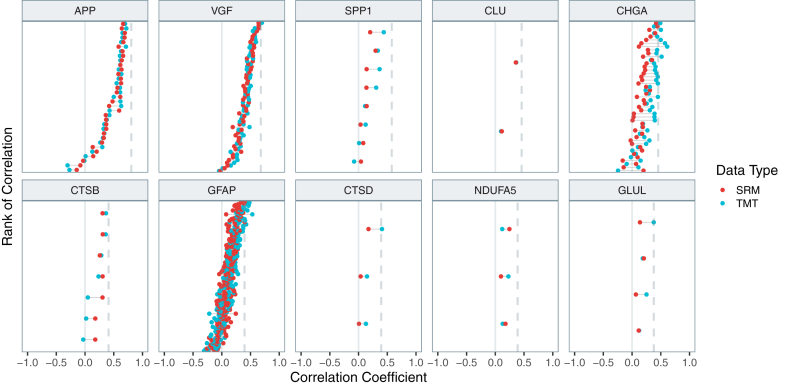
**Correlation of TDP proteoforms with the parent protein BUP (SRM and TMT) measurements.** Individual points represent proteoforms of a given protein. Plots are shown for proteins that were present in all three sets of data, had high correlation (R > 0.35) between the two BUP datasets, and had more than one proteoform in TDP data. Proteoforms are ordered on the Y-axis according to their average correlation to the BUP datasets. The *dashed line* reflects the cross-BUP (SRM/TMT) correlation value for a given protein.

Calculating correlation between global protein abundance derived from bottom-up and intact proteoform measurements from top-down is not straightforward, since given our top-down data, there are seven proteoforms per protein on average. Therefore, we evaluated the correlations of all proteoforms with both types of BUP data (Fig. 3). Overall, the TDP-BUP abundance relationships were positively correlated. For most of the proteins, there is at least one proteoform with the same or higher correlation to the BUP data compared to the reference value based on the two BUP data sets. Thus, despite the complexity of the protein posttranslational processing and bias of the TDP method towards low-MW species, there is evidence that, overall, the data agrees with conventional proteomic approaches.

### Proteoforms Linked to Neuropathologies and Cognitive Decline

**Fig. 4:**
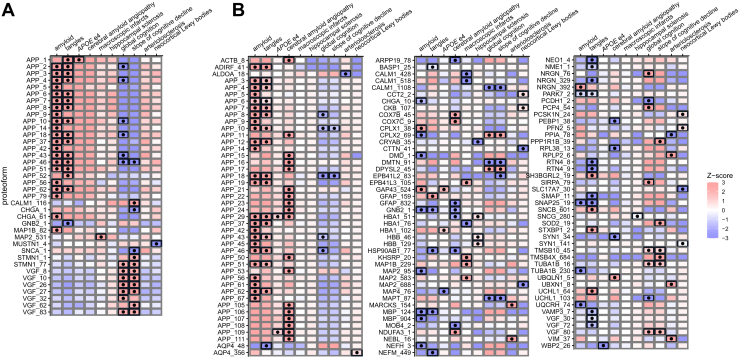
**Statistical associations.** Punchcard heatmaps of statistical associations between proteoforms and cognitive/pathological variables using (*A*) intensity-based measurements and (*B*) spectral counts. For label-free intensities, associations were modeled using linear regression while spectral counts associations were modeled by a quasi-Poisson generalized linear model. In both modeling approaches, abundances were adjusted using postmortem interval as a covariate. *Black* dots represent statistically significant associations (Benjamini–Hochberg adjusted *p*-values <0.05). Proteoforms are labeled with their gene of origin, followed by an arbitrary number assigned based on clustering.

Next, we tested the association between proteoform relative abundances and clinicopathological variables. For the statistical analysis, we utilized linear and quasi-Poisson modeling approaches for the intensity and spectral counting data, respectively (33). Intensity data was corrected for batch effects prior to statistical testing. In case of spectral counting data, the batch effect was added as a covariate into the statistical models. We also accounted for postmortem interval in both types of measurements, as it was significantly associated with a substantial number of proteoforms: 200 and 164 proteoforms for intensity-based and spectral counting approaches, respectively. The tested variables included tangle density, amyloid load, presence of Lewy bodies in the neocortex, hippocampal sclerosis, cerebral infarcts, cerebral amyloid angiopathy, *APOE* ε4 allele status, cognition level, slope of cognitive decline, and the slope of cognitive decline adjusted for known Alzheimer's disease and related dementias neuropathologies. The summary heatmap in Figure 4 demonstrates the associations discovered by testing both types of quantitative data. In total, 41 proteoforms in intensity-based quantification and 125 in spectral counting–based quantification showed a statistically significant relationship with at least one variable. The overlap between the two approaches is 18 proteoforms. Many of the significant proteoforms are derived from proteins that have been linked to AD (https://agora.adknowledgeportal.org/about) (66). This includes proteins such as growth-associated protein 43 (GAP-43), MAP1B, MAP2 (67), alpha-synuclein (SNCA), ACO2, SYN1, stathmin (STMN1), VGF (68), PACSIN1 (69, 70, 71), and VAMP3 (72, 73, 74).

### Heterogeneity of Aβ Proteoforms

**Fig. 5:**
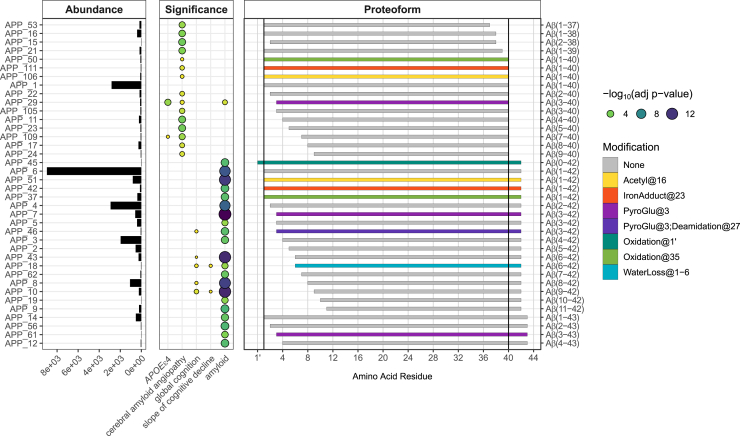
**Differential association of amyloid beta proteoforms between amyloid plaques and cerebral amyloid angiopathy.***Leftmost* panel contains bar plots showing the spectral count abundance for each amyloid beta proteoform. *Middle* panel displays the significance of the association between each amyloid beta proteoform and several clinicopathological or genetic variables, including *APOE* ε4 status, cerebral amyloid angiopathy pathology, last visit global cognition score, person-specific rate of cognitive decline adjusted by age, sex, and education, and overall amyloid level (amyloid). The degree of significance is indicated by the circle size and color. The *rightmost* panel maps every amyloid beta proteoform to an APP reference sequence (*dark gray* bar) and colors them by the type of PTM identified. Amyloid beta proteoforms are sorted by ascending C-terminal amino acid ending position, followed by ascending N-terminal amino acid starting position.

We quantified 37 and 49 Aβ proteoforms using intensity and spectral counting approaches (35 in common), respectively. The most abundant Aβ proteoforms were unmodified Aβ_1-42_, followed by Aβ_2-42_ and Aβ_1-40_ (Fig. 5). The observed species had truncations and extensions of the C-terminus and predominantly truncations from the N-terminus, relative to the canonical Aβ_1-40_ species. The truncations from N- or C-termini were mutually exclusive with only one exception of low abundance Aβ_2-38_ (APP_15) species truncated from both termini.

**Fig. 6:**
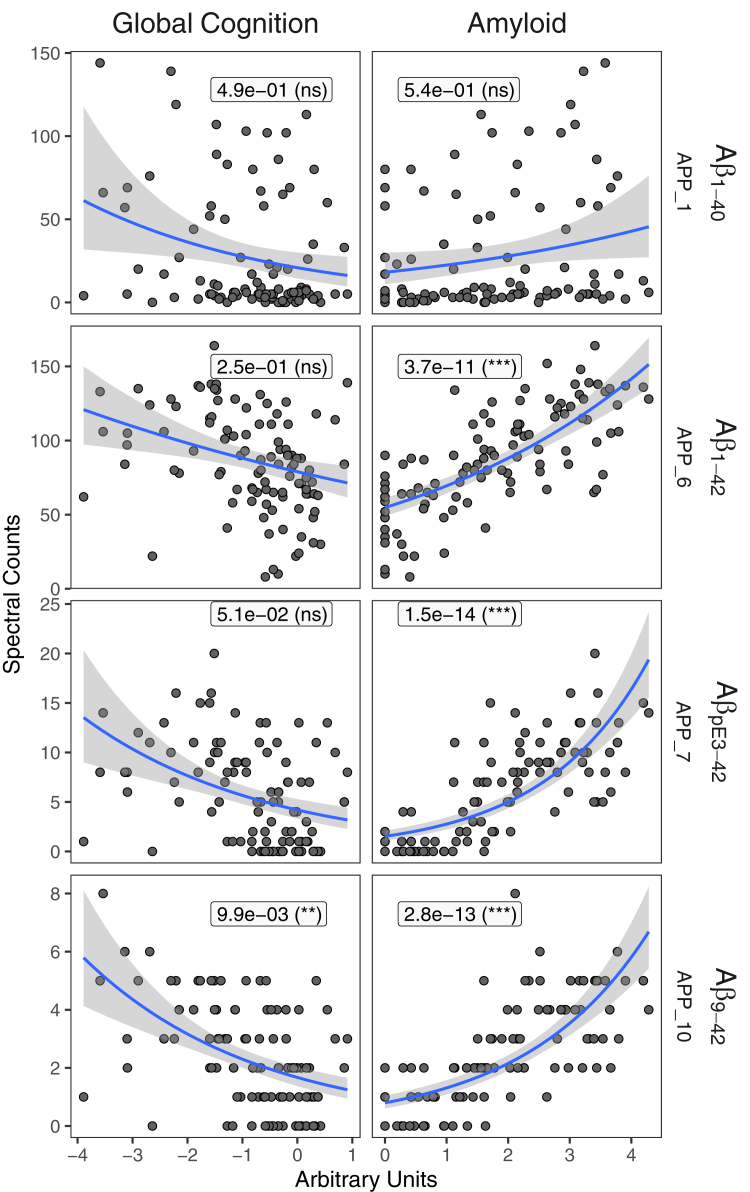
**Association of selected Aβ proteoform abundances (y axis) with the global cognition level and amyloid plaques load (x axis).** Proteoform abundances were quantified using spectral counting and modeled using a quasi-Poisson approach. The estimate of the mean and the corresponding standard error are shown as a *blue* line and shaded area. For the purpose of visualization, the fitted model does not include the batch effect. The Benjamini–Hochberg adjusted *p*-values (ns > 0.05, ∗ < 0.05, ∗∗ < 0.01, and ∗∗∗ < 0.001) are shown for the full model that factors in batches and are the same as in Figure 4.

We observed several PTMs on Aβ as well. The most abundant PTM-bearing Aβ proteoforms included pyroglutamination of Glu3 (Aβ_pE3-42_), acetylation of Aβ_1-42_ at Lys16, and oxidation of Aβ_1-42_ at Met35. We found the Aβ_pE3-42_ (APP_7) abundance to be most associated with amyloid plaque relative to all proteoforms quantified in this analysis including canonical Aβ_1-42_ (APP_6, Fig. 6). Interestingly, the other main species Aβ_1-40_ (APP_1) abundance is not significantly associated with amyloid plaques or any other clinicopathological variable.

Overall, Aβ proteoforms can be divided into two major groups and one subgroup based on the starting and ending positions. The longer forms with C-terminal positions greater than 42 are associated with amyloid load, while shorter forms ≤40, with exception of Aβ_1-40_, demonstrate association with CAA. All the CAA-associated proteoforms were truncated either from the N- or C-terminus relative to the 1st and 40th amino acid, respectively. Canonical Aβ_1-40_ proteoforms were not statistically significantly associated with CAA unless they were bearing PTMs such as oxidation of Met35 or acetylation of Lys16. However, in these cases, the association was not as strong as for N- or C-terminus truncated species (*e.g.* Aβ_1-38_ or Aβ_4-40_). Finally, there is a small subgroup of Aβ_x-42_ species with N-terminal truncations from positions 3 to 9 that demonstrate particularly strong association both with the neuropathology and cognition (*e.g.* APP_10, Fig. 6).

### Association of VGF Proteoforms with Cognitive Decline

**Fig. 7:**
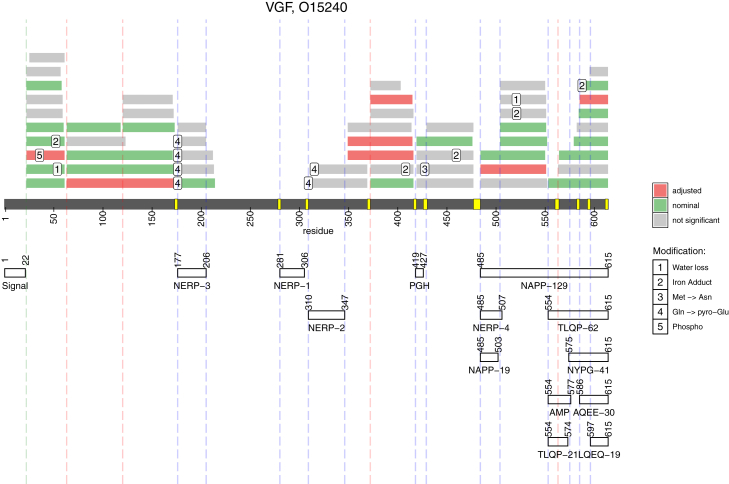
**Proteolytic cleavage map of VGF.***Middle gray* bar and residue numbers indicate the full-length VGF protein. *Yellow* markings along the protein sequences denote the di-basic sites that are the putative cleavage sites for proprotein convertases. Annotation of the known VGF neuropeptides is shown below this residue labeling (*white bars*). The VGF proteoforms quantified in this study are shown in the upper half and colored according to significance threshold (nominal significance is shown in *green*, adjusted significance is shown in *red*, not significant forms are shown in *gray*). Observed posttranslational modifications are numbered and annotated at their localized amino acid position. Vertical *dashed blue lines* indicate known cleavage sites while *dashed redlines* show the previously unknown cleavage sites. The single *dashed greenline* indicates the cleavage site for the signal peptide.

VGF is another protein that has been gaining attention with regards to its association with AD (68, 75, 76). Notably, it is expressed as a polypeptide that is proteolytically processed by proprotein convertases (77, 78). The observed VGF fragments in our study align with known cleavage sites and previously annotated neuropeptides (77) (Fig. 7). Mapping of the cleavage sites revealed signal peptide cleavage along with 13 previously described internal cleavage sites and four newly characterized cleavage sites. Notably, cleavages were observed for all dibasic sites.

Association of VGF fragments with cognitive decline was not limited to a particular neuropeptide or a region of the VGF sequence. The seven (out of 55 quantified) statistically significant proteoforms are scattered throughout the entire sequence. If no correction for multiplicity of hypothesis testing is applied, then the number of significant proteoforms goes up to 27. However, even in this case, there is no regional dependence of VGF association with cognitive decline (Fig. 7).

### Co-abundance Clustering Analysis

#### Individual Cluster Patterns

**Fig. 8:**
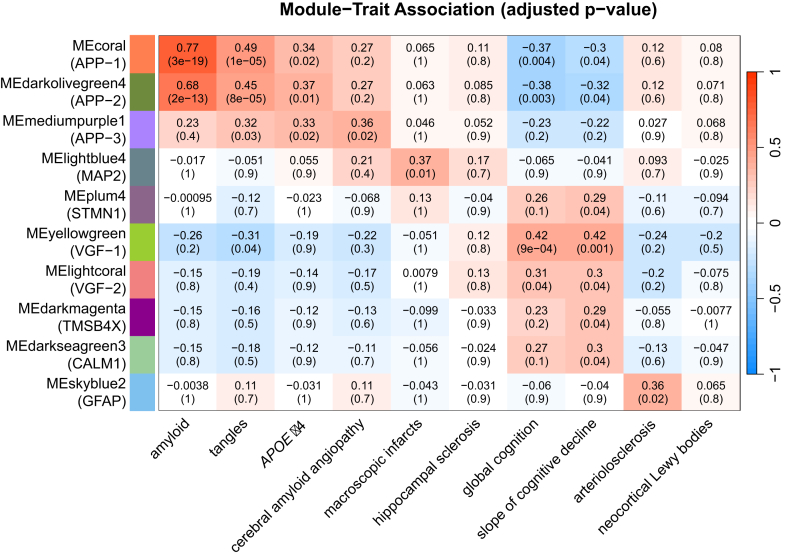
**Heatmap of the significant associations of WGCNA modules' eigengenes with selected clinical and pathological variables.** The amplitude and directionality of the correlation in color-coded in *blue*-*white*-*red* gradient and is shown in each cell. The adjusted *p*-values, reflecting the significance of the association, are shown in *parentheses*.

To group the proteoforms into co-abundance modules, we applied a widely used WGCNA clustering tool. This resulted in 95 modules, with on average 63 proteoforms per module with sizes ranging from 5 to 3267 proteoforms. The module centroids were correlated with the selected phenotypes. Overall, 10 modules had significant associations with at least one of the traits (Fig. 8 and Supplemental Table S4). For each of the 10 significant modules, we selected the representative gene with the highest number of proteoforms in the module. Some genes, specifically APP and VGF, were representative in more than one module. Furthermore, several modules were represented by proteoforms of predominantly (>85%) a single parent gene: APP-1, APP-2, APP-3, VGF-1, VGF-2, and STMN1. Three additional modules had >50% proteoform representation of a single gene (GFAP, CALM1, and MAP2).

The most significant association is between APP-1 (or “coral”) module and amyloid load (adjusted *p*-value 4 × 10^−19^). It contains Aβ_1-42_ and other Aβ proteoforms ending at the same C-terminal position but recessively shortened at their N-terminal starting positions. The association is exceptionally statistically strong because these Aβ_x-42_ essentially constitute amyloid plaques. APP-2 (or “darkolivegreen4”) is another module that contains similar Aβ proteoforms with the same pattern of associations across the phenotypes as APP-1, but with weaker statistical significance. We did not notice any differences between the APP-1 and APP-2 modules in the size of the proteoforms, their starting N- and ending C-termini positions, or PTMs. However, APP-2 module proteoforms showed noticeably (∼30-fold) lower in abundance (Supplemental Fig. S9). The third APP module, APP-3 (or “mediumpurple1”) had a clearly different profile of association with phenotypes. It was not associated with amyloid plaques but showed significant association with CAA. The APP-3 cluster essentially captured the Aβ species truncated at the C-terminus that were described above (Fig. 5).

The split of VGF into two modules is similar to the split between APP-1 and APP-2. The VGF-1 (or “yellowgreen”) is a module composed of less abundant species and overall has stronger association with cognition. The VGF-2 (or “lightcoral”) cluster contains more abundant VGF proteoforms and shows a similar behavior with a weaker association with cognition. No differences were found between the two VGF modules in terms of PTMs, fragments’ length, or position within the protein sequence.

Modules “plum4,” “darkmagenta,” and “darkseagreen3,” represented by the genes STMN1, TMSB4X, and CALM1, respectively, show association profiles similar to the VGF modules. The significant associations, like in case of VGF, are primarily contained within the variables reflecting cognitive metrics (Fig. 8). Given that the directionality of the associations is also the same, they may represent the same underlying process related to VGF. Additionally, the “darkmagenta” cluster is enriched with methionine oxidations. Notably, 14 of the 27 proteoforms within the cluster contain mono- or di-oxidation events. These oxidations are distributed across nine out of 16 genes represented in the cluster. By Fisher's exact test, this enrichment is highly significant (*p*-value = 1 × 10^−14^) in comparison to the global data in which oxidation represents <5% of observed modifications (Fig. 2*D*). While there is currently no established causal relationship between global protein oxidation and progression of AD (79, 80), methionine oxidation does have broad consequences for protein structure and activity (81, 82).

Module “lightblue4”, associated with gross chronic infarcts, consists predominantly of MAP2 proteoforms, followed by DYNLL1, MAP1A, and SYNGAP1. MAP2 itself is a cytoskeletal protein localized in the dendrites, while other members of the module are also annotated as cytoskeletal or dendritic proteins. The “skyblue2” module, associated with arteriolosclerosis, is dominated by GFAP proteoforms, followed by proteoforms derived from AQP4, MARCKS, and SIRPA. The latter three proteins are either membrane, trans-membrane, or anchored to the cytoplasmic membrane.

## Discussion

### Introduction

We quantified 6124 and 7510 proteoforms, mapped to 836 and 952 proteins, using intensity- and spectral counting–based approaches, respectively. Thus, for each protein, we observed approximately 7 to 8 proteoforms on average. This study also provides deep characterization of brain proteoforms, capturing unique PTMs such as phosphorylation, glycosylation, and pyroglutamylation. Our data suggests three quarters of all proteoforms that start at the N-terminus (with or without methionine excision) were N-terminally acetylated (Fig. 2*D*). This is in agreement with the high frequency of N-terminal acetylation in the human proteome (83).

Both types of quantitative data, proteoform intensity-based abundances and spectral counts, were correlated with clinical and neuropathologic variables relevant to cognitive decline and AD. This approach enabled a detailed look at proteoforms within a given gene with regards to their role in AD. We identified associations involving canonical AD-related proteins such as amyloid beta, MAPT, and VGF, as well as new proteoform-disease correlations that have not been well characterized, such as with GAP-43 and stathmin. In the case of MAPT (tau), we observed many truncated proteoforms; however, only one was linked to general neuropathology. This does not necessarily indicate a weaker association of tau with neuropathology but instead is a limitation of the method as full-length tau (46 kDa for the 2N4R isoform) is above of the typical molecular weight range for TDP experiments. Thus, capturing pathology-relevant species of tau would require either targeted acquisitions (84, 85), partial digestion or middle-down proteomics approaches (86, 87, 88, 89), or alternative data acquisition methods (90).

### Considerations for Further Top-Down Quantitative Studies

Unlike bottom-up proteomics, the field of quantitative TDP is still in its early stages. Various approaches have been explored, including spectral counting (19), MS1 intensities, extracted ion chromatograms (47), or even isobaric tag labeling (91). Our initial goal was to use MS1 intensities for quantification. To facilitate this, we developed TopPICR (17), a tool that aligns datasets and performs match-between-runs analysis—a feature first implemented in MaxQuant (92).

Spectral count data is inherently present in any data-dependent acquisition dataset, requiring no additional experimental effort. As a result, both types of quantitative data can be generated. One key challenge in TDP data analysis is the ambiguity in PTM localization, which can lead to partially characterized proteoforms being assigned different identities across datasets. To address this, we strongly recommend aligning datasets and grouping proteoforms based on their mass and elution time—a process performed by TopPICR—even when spectral counting is used for quantification. Without this step, reports will contain an inflated number of proteoforms and a high degree of missingness or zero counts.

In this study, spectral counting provided broader coverage of proteoforms, likely due to the more stringent data completeness requirements for label-free statistical testing. However, studies involving larger number of samples offer greater statistical power, thus enabling the use of less statistically sensitive spectral counting approaches (e.g., quasi-Poisson). Conversely, for smaller datasets (N ≤ 10), MS1-based label-free quantification, particularly when combined with moderated statistical tests (*e.g.*, limma), may be a more effective approach.

### APP

#### Introduction

Aβ proteoforms provided the strongest associations to Alzheimer's clinicopathological variables across both spectral counting and intensity-based analyses. More notably, our study identified the largest number of human brain Aβ proteoforms to date in a single study, including four extended Aβ_x-43_ proteoforms that typically cannot be observed in BUP LC-MS/MS studies due to the extreme hydrophobicity of the C-terminal tryptic peptide (93). Part of this depth can be attributed to the HFIP extraction method developed within this study, which can efficiently solubilize Aβ while at the same time removing potential contaminating proteoforms from the supernatant. Overall, the catalog of Aβ proteoforms found in this study also agrees with a recent TDP studies using human postmortem samples (94) and the 5xFAD mouse model (47). In particular, all three datasets identify a variety of N- and C-terminally truncated proteoforms in addition to the most abundant proteoforms Aβ_1-42_ and Aβ_1-40_.

#### HFIP as an Aβ Solubilizing Agent

Aβ solubilization is a topic of a broad and persistent interest. The “gold standard” approach for Aβ solubilization is treatment with 70 to 100% FA (41, 42, 43). However, this method is not ideal for TDP, as it can introduce multiple formylation modifications on a proteoform as well as artificial cleavages, complicating downstream analysis (44, 95). To overcome this challenge, we surveyed alternative solubilization strategies with strong chaotropic effects and limited interference with LC-MS instrumental analysis and data interpretation. Alternatives that principally fit into this category were HFIP, guanidinium, and urea. HFIP has been shown to effectively dissolve synthetic Aβ_42_ peptides into monomeric forms by disrupting β-sheet structures (96, 97). It has also been used as a mass spectrometry–friendly solvent in a recent top-down study (98). While it remains to be demonstrated that HFIP “fully” solubilizes human brain-derived amyloid plaques, the measured Aβ proteoforms are significantly associated with expected clinicopathological variables. First, Aβ_X-42_ proteoforms correlate with plaque content measured *via* histological techniques; even if these species do not originate directly from plaques, this implies that they are at least plaque markers. Second, the abundances of HFIP-extracted Aβ species align well with those from another study using 4M guanidine to resolubilize pelleted, “insoluble” Aβ fractions (Supplemental Fig. S2). Third, Aβ proteoforms were among the most abundant in our dataset, ranking third in spectral count–based abundance estimates, alongside MBP and GFAP. This suggests efficient extraction, with approximately 3.5% of all spectra assigned to Aβ proteoforms alone.

We acknowledge that the evidence for HFIP's ability to fully solubilize Aβ plaques is circumstantial at best, and some Aβ proteoforms may not have been captured. Nonetheless, there is a strong statistical association between HFIP-soluble Aβ species and both plaques and CAA. Notably, the differential association of Aβ42 species with plaques *versus* Aβ38 and Aβ40 with CAA is well-defined. Thus, we believe, HFIP is a solubilizing agent worth considering for future proteomics Aβ species studies.

#### Differential Association of Aβ Proteoforms in Human and Mice

The Aβ proteoforms identified in this study could be divided into two groups based on their association with either amyloid plaques or CAA. The two groups could also be distinguished by the ending C-terminal residue, with CAA-linked proteoforms ending at or before the 40th amino acid and plaque-linked proteoforms ending at the 42nd and 43rd amino acid. Interestingly, the WGCNA modules corresponding to longer Aβ_x-(42,43)_ (“coral” and “darkolivegreen4”) and shorter Aβ_x-(37-40)_ species (“mediumpurple1”) have also been observed in the mouse 5xFAD model as “Group1” and “Group2” proteoforms. However, in the case of the 5xFAD model study, the distinction between the clusters was based on profiles across age groups and soluble/insoluble fractions (Fig. 4 in ref (47)). We can be cautiously optimistic that 5xFAD model reproduces the differences in the behavior between the Aβ_x-(42,43)_ and Aβ_x-(37-40)_ forms. If the group2 Aβ species have statistically significant association with CAA in the 5xFAD mouse, like we observed in this human study (Fig. 5), that would further validate the model for studying the differences in the Aβ processing, accumulation, and clearance pathways. Note, that we did not robustly observe Aβ proteoforms present in the mouse group 3 cluster. There are a few potential reasons for this. One is that we utilized a 3K MWCO filtration step during sample preparation which would remove smaller proteoforms, such as those in the mouse group 3 cluster. Due to LC-MS duty cycle constraints, we also only analyzed three FAIMS CV values between −50 V to −30 V. As FAIMS CVs are correlated with proteoform size (15), observation of shorter Aβ proteoforms such as Aβ_1-25_ would likely require analyzing CVs between −50 V and −70 V or lower.

#### Cerebrovascular Amyloid

Although several prior studies have observed C-terminally truncated Aβ proteoforms within cerebrovasculature (99, 100, 101, 102), they have been limited in the number of subjects and Aβ species examined. Another peculiar observation was made during an early TDP characterization effort of Aβ species (94). The sample preparation method in that study involved separating parenchyma from blood vessels. To the authors surprise, they did not observe any proteoforms ending at the 37th or 38th residue. Given our results, that these short Aβ forms are more closely associated with CAA, the most likely explanation is that the sample was depleted of these short forms by removing the vasculature. However, the authors did detect N-terminally truncated Aβ_x-40_ species, several of which were found to have associations with CAA according to our data. Taking these results together, the data imply a regional difference in Aβ processing. In summary, Aβ species can be thought of at least as two distinct groups, one consists of C-terminally extended forms (Aβ_x-42,43_) associated with dementia-related amyloid plaques, while the other consists of C-terminally truncated forms (Aβ_x-(37-40)_) associated with CAA-associated deposits restricted to the vasculature.

#### Effect of N-Terminal Truncations

Analysis of the spectral counting data indicates a subset of Aβ_x-42_ species with N-terminal truncations that significantly associate with cognitive level and the speed of its decline (Fig. 5). The relevant truncations span from third (pyro-Glu modified) to ninth amino acid. The pattern of Aβ_x-42_ proteoforms most significantly associated with cognition and amyloid plaques are different depending on the N-terminus, indicating an additional or independent process that involves N-terminal truncations. Unlike gamma-secretase, beta-secretase cleaves specifically between the 671 and 672 residues of APP sequence (103). Thus, the N-terminal truncations are most likely produced by other proteases. Our data suggests that N-terminally truncated species, for example, Aβ_9-42_, might be a stronger biomarker of the clinical manifestation of AD compared to the canonical Aβ_1-42_ form. Thus, it may serve as a better engagement marker for drug clinical trials if captured in blood plasma. Given the rise of assays that are based on Aβ proteoform ratios (104, 105, 106, 107), it may be of interest to investigate the performance of Aβ_9-42_, Aβ_1-42_, or Aβ_1-40_ as a correlate or predictor of cognitive decline rather than overall Aβ plaque pathology.

#### Pyro-Glu Aβ

We found pyroglutamate-containing Aβ_pE3-42_ to be most strongly associated with amyloid plaques, which aligns with prior evidence demonstrating the pyroglutamyl form of Aβ has a much higher propensity for aggregation relative to Aβ_1-x_ forms (49). This increased aggregation can be attributed to the loss of two charges, the N-terminal amine and carboxyl from the side-chain of the glutamate, thus reducing overall solubility of the species (108). Cyclization of N-terminal Glu into pyro-Glu can occur as a spontaneous chemical reaction (109) or as an enzymatic reaction catalyzed by the enzyme glutaminyl cyclase (110, 111). Indeed, recent studies have demonstrated that Aβ_pE3-x_ requires enzymatic conversion by glutaminyl cyclase (112), which has led to consideration of these enzymes as therapeutic targets (113). Our results indicate that, despite its low abundance, Aβ_pE3-42_ has the strongest association with amyloid plaques.

#### Context of Aβ Proteoforms in Clinical Applications

Considering the diversity of Aβ proteoforms described in this study and their differential pathological associations, the impacts of this heterogeneity on anti-amyloid therapies and immunotherapies have likely been severely underestimated in clinical trials as amyloid burden is often nonspecifically assessed by PET imaging (114, 115). Although recent trials have started to adopt plasma biomarkers such as Aβ_1-40_ and Aβ_1-42_ (116, 117), our data suggests that more proteoforms need to be assessed in parallel to fully understand the impacts of AD therapies (114, 118).

For example, what proteoforms are targeted by anti-Aβ monoclonal antibodies and how these antibodies impact amyloid plaques, CAA deposits, and potentially other Aβ proteoforms remains unclear, resulting in unpredictable clinical outcomes (119). One example is ponezumab, a therapeutic antibody that was described as recognizing the C-terminus of Aβ_1-40_ and required a free carboxy terminus at position 40 for binding (120). Ponezumab was reported to reduce deposition of amyloid in cerebral blood vessels during preclinical research and therefore considered as a treatment for CAA, but was discontinued after showing trends opposite expected outcomes in phase two clinical trials (121, 122). Our results demonstrate that Aβ_1-39_, Aβ_1-38_, and Aβ_1-37_ (and several modified Aβ_x-40_ proteoforms) are more closely associated with CAA than Aβ_1-40_. Therefore ponezumab, which lacks binding to Aβ species truncated from position 40, should not be expected to clear all the proteoforms related to CAA pathology.

Another example is donanemab which targets pyro-Glu Aβ_pE3-42_ and has shown positive results in clinical trials (123). While we confirmed the importance of the pyro-Glu Aβ_pE3-42_ based on having the strongest correlation with pathology, which agrees with the rationale behind the development of donanemab (123), the low abundance of pyro-Glu AB_pE3-x_ (relative to other Aβ proteoforms identified in our study) suggests looking for other anti-amyloid treatments with affinity to more prevalent and more impactful Aβ proteoforms such as Aβ_9-42_.

Finally, a recently developed antibody-based drug aducanumab (aduhelm) (124) recognizes the Aβ domain 3 to 7 (EFRHD) amino acids (125) and should theoretically bind to a wide range of proteoforms. Its efficacy in humans has been demonstrated using a surrogate endpoint based on PET imaging that does not distinguish parenchymal from vascular Aβ deposits. Although it was demonstrated that aducanumab more prominently binds parenchymal Aβ deposits in Tg2576 transgenic mice (124), major safety concerns arose, including hemorrhages in human subjects, specifically amyloid-related imaging abnormalities with vasogenic edema, which clearly point to detrimental changes in the vasculature. This leads to two possible explanations; that aducanumab damages the vasculature by removing vascular Aβ deposits or conversely exacerbates CAA by transporting parenchymal Aβ to the vasculature (126, 127). We can envision that the TDP approach, capable of resolving dozens of Aβ proteoforms, will be an indispensable tool for understanding the effect of aducanumab on Aβ clearance and brain swelling/hemorrhage complications.

### VGF

VGF, also known as secretogranin VII, belongs to the granin family of neuropeptides. It is posttranslationally processed into neuropeptides with various biological activity (reviewed in (77, 78, 128, 129)). Several of them have been implicated in AD and other neurodegenerative or mental disorders. For example, TLQP-21 modulates microglial function (76), TLQP-62 reduces Aβ plaque burden and disease-associated microglial activation (68), and other VGF peptides have been noted as decreasing in abundance in the brain, CSF, or blood plasma in AD or Parkinson's diseases (Table 2 in (78)). It is not trivial to reconcile the fact that a variety of VGF-derived neuropeptides have similar associations with AD and yet have different sequences and thus most likely different functions. Accordingly, a recent study targeting these different proteoforms with tryptic peptides on a large number of subjects (N = 1020) found that the association with AD and neuropathologies is not specific to a particular peptide or region of VGF (75).

Out of the 55 VGF fragments quantified in our study, 27 were nominally significant in the association with cognitive decline while seven remained significant after adjustment of the *p*-values for multiplicity of hypothesis testing (Fig. 7). Importantly, statistically significant proteoforms could not be traced to a specific region of the VGF protein. Therefore, our results and prior data indicate the association of VGF with AD is not unique to a single VGF neuropeptide or a specific region of the protein. There may be two explanations that form two, but not mutually exclusive hypotheses. One hypothesis is that, indeed, numerous VGF neuropeptides are necessary for maintaining cognition. An alternative hypothesis is that there is no differential regulation of the VGF-derived peptides. That is, once the full-length VGF protein is synthesized and cleaved by prohormone convertases, there is no differential regulation between the produced peptides, thus they are mutually correlating and have indistinguishable association with cognition.

### STMN1

Stathmin, an 18 kDa tubulin-binding protein that inhibits microtubule assembly (130), was observed to have 17 quantifiable proteoforms, two of which were positively correlated with the slope of cognitive decline individually. A larger number of STMN1 proteoforms formed a cluster (“plum4”) that had a similar profile to VGF. The associated proteoforms encompassed the full-length protein sequence, shortened fragments, and unlocalized ∼15 Da modification which may be indicative of a single-nucleotide variant. A few reports have shown that stathmin is decreased in AD patients and adult patients with Down syndrome, and stathmin-dependent changes in microtubule stability are essential for memory stability (131, 132, 133).

### GAP-43

GAP-43, also referred to as neuromodulin, is an intracellular growth-associated protein that is associated with nerve growth. An N-terminally truncated, phosphorylated GAP-43 proteoform (GAP43_524, phosphorylated ambiguously at serine 150, 153, or 154) was found to have an inverse relationship with amyloid plaque burden, contrary to previous reports on the full-length protein (134). Phosphorylation of GAP-43 is known to play a role in axon growth and synaptic plasticity (135), and we also observe this GAP-43 proteoform is associated with reduced cognitive decline.

## Conclusions

This TDP approach has provided a unique perspective to investigate the proteome with respect to AD and associated pathologies. The global approach for the analysis of intact proteins has decisively established the clinicopathological relevance of two major types of Aβ—one associated with amyloid plaques and the other with similar types of deposits, known as cerebral amyloid angiopathy, that are localized to the brain vasculature. We have also discovered associations between N-terminal truncation state of Aβ_42_ and cognitive decline. Considering these results, we believe the TDP approach will be important for guiding future anti-Aβ therapies. Furthermore, we have confirmed that VGF does not have a unique neuropeptide fragment associated with cognitive level or its decline. Finally, numerous other proteoforms had significant associations with either cognition or pathology; however, their role in neurodegeneration is less clear and will be a focus of further investigations.

## Data Availability

All raw files have been deposited into the MassIVE data repository and can be accessed *via* accessions MSV000093728 and MSV000093727 for the human and mouse components of the study, respectively.

## Supplemental data

This article contains supplemental data (47).

## Conflicts of interests

The authors declare that they have no conflicts of interests with the contents of this article.
