# Supplementary material for: Discovery of Proteoforms Associated With Alzheimer's Disease Through Quantitative Top-Down Proteomics
Source: Mol Cell Proteomics. 2025 May 5;24(6):100983. doi: 10.1016/j.mcpro.2025.100983 (PMC12173667; doi:10.1016/j.mcpro.2025.100983)
Supplement: Supporting_data [file mmc4.docx]

# Supplementary Figures


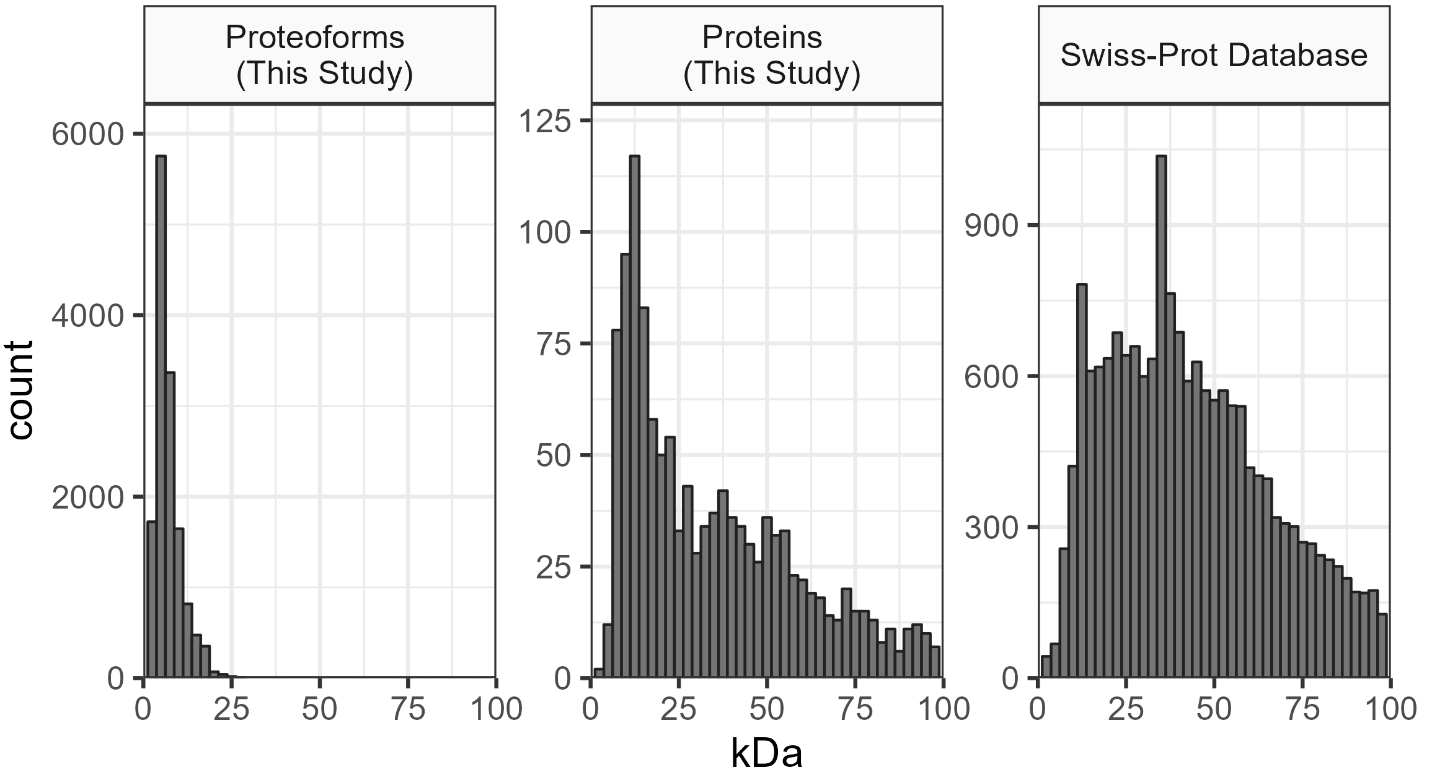


**Supplementary Figure S1**. Distributions of the molecular masses of the unique proteoform species identified in this study, their parent proteins and all the proteins in the Swiss-Prot database. Histogram bin width is 2.5 kDa.


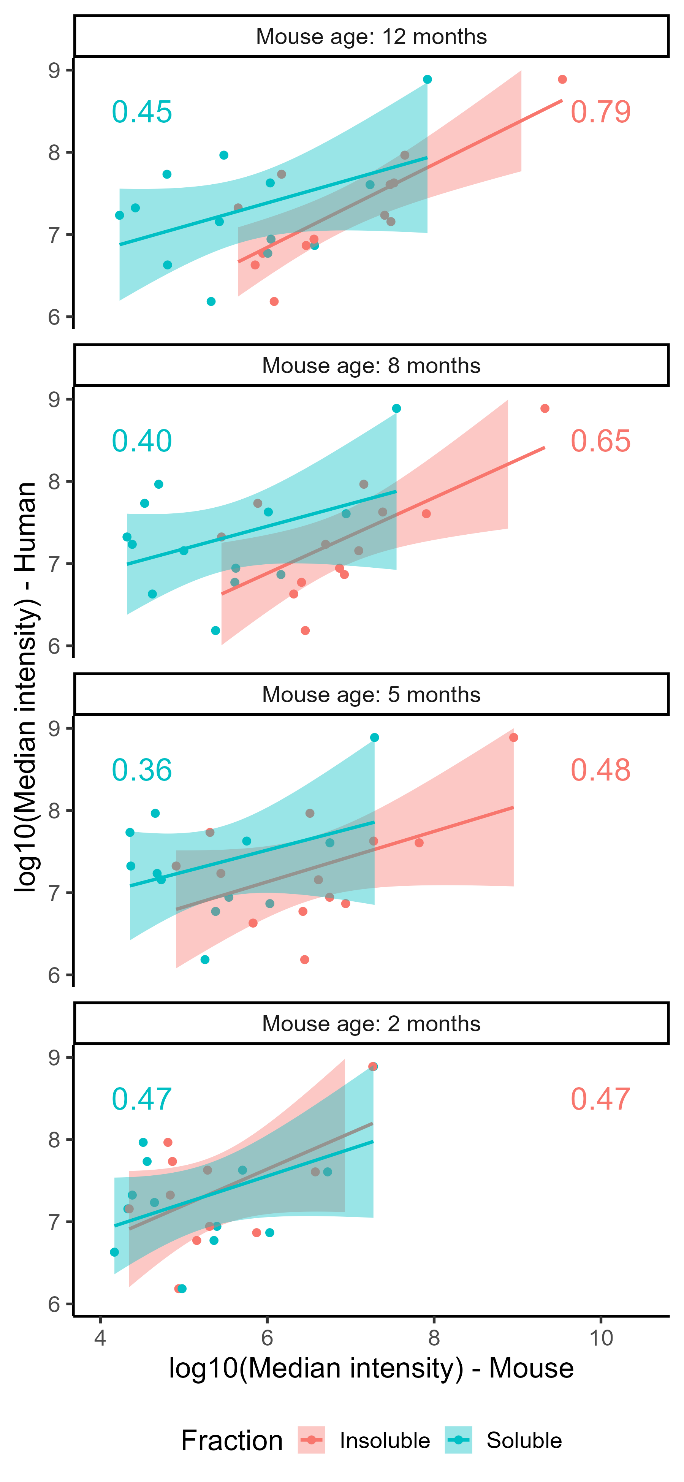


**Supplementary Figure S2.** Intensities for proteoforms of Aβ were compared between this analysis (y-axis) versus prior analysis of 5xFAD mice (x-axis)^47^. Intensity is defined as the median intensity of a given proteoform across all subjects, with mouse samples grouped by age. Color fill of the regression lines and the confidence intervals denote the insoluble (pink) or soluble (blue) fraction. Individual panels denote the age of the mice, with N=6 mice per age group. Pearson correlations values for insoluble and soluble fractions are denoted in the panels.


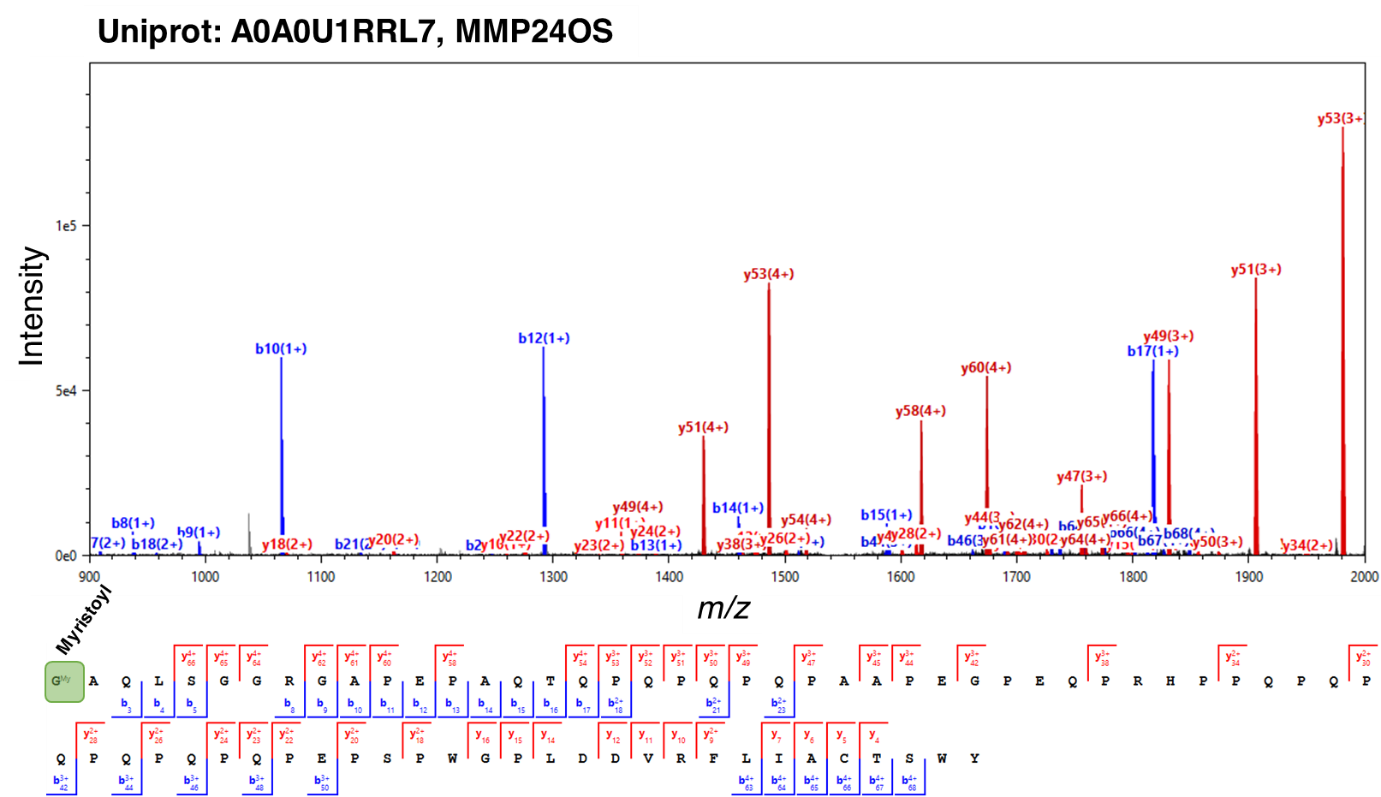


**Supplementary Figure S3**. TDP spectrum confirming the N-terminal myristoylation of MMP24OS. Top panel displays the annotated fragmentation spectrum with most peaks matched to b-type and y-type fragment ions. Lower panel displays MS2 sequence coverage map. Blue wedges indicate the matched b-type ions from the N-terminus and red wedges indicate the y-type ions from the C-terminus along the protein sequence.


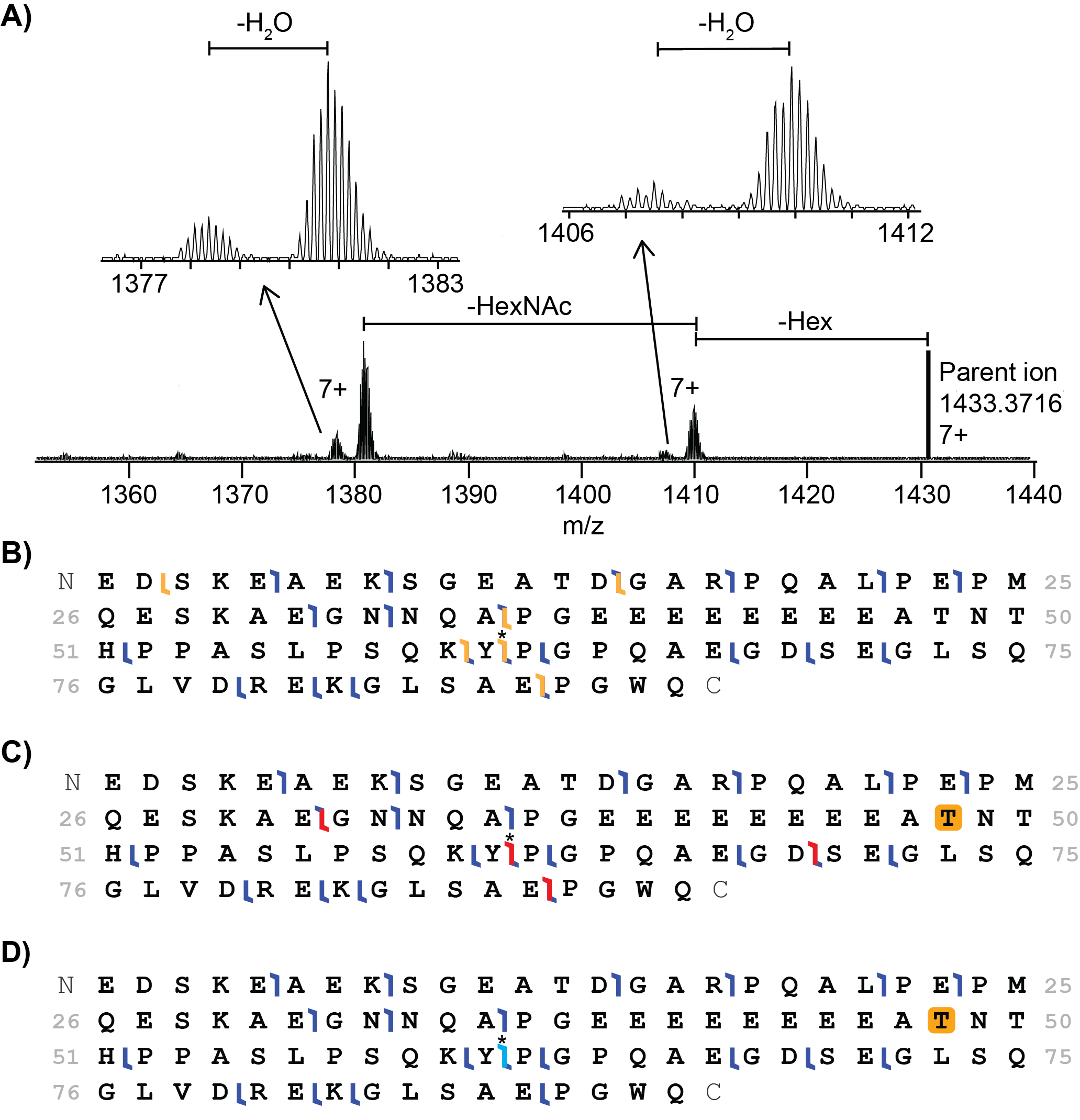


**Supplementary Figure S4**. MS2 data (CID = 35) of glycosylated (HexHexNAc) CHGA (Accession: P10645). **A)** Inset of MS/MS spectra showing parent ion location (1433.3716 m/z, 7+) and subsequent neutral loss forms where Hex or HexHexNAc are ejected from the intact parent ion. Assigned fragment ions are also shown for three searched proteoforms including the **B)** unmodified sequence, **C)** HexNAc modification at T181, or **D)** HexHexNAc modification at T181. Unique fragment ions are denoted in either gold, red, or light blue, with gold and red fragment ions exhibiting either partial or full neutral loss of the HexHexNAc modification. Notably, B61 (*) exists as three forms with either the full HexHexNAc modification and subsequent neutral loss.


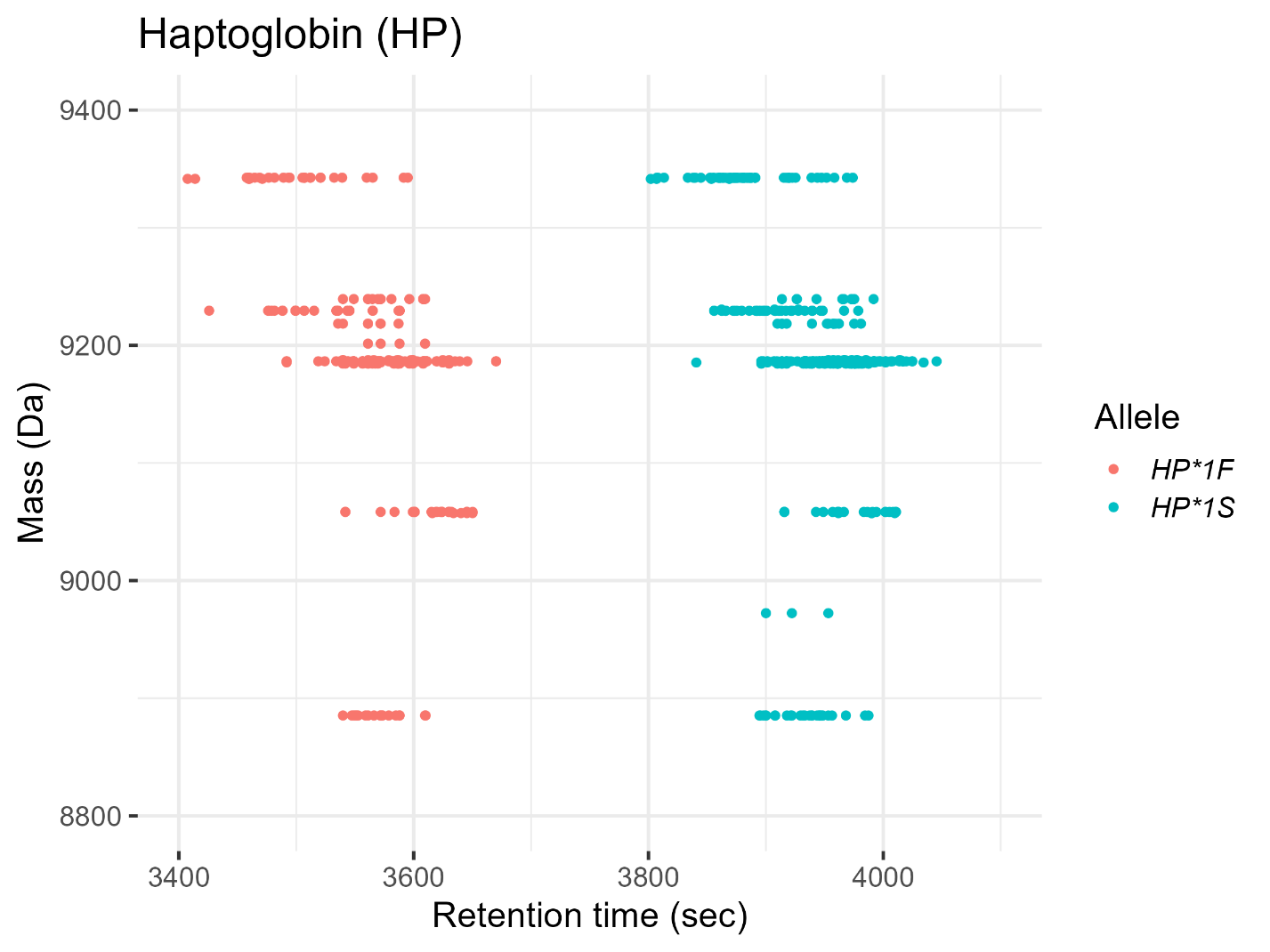


**Supplementary Figure S5**. LC-MS clusters of two isomeric haptoglobin allele variants. Points represent assigned masses (Da) versus retention time (sec). Alleles are denoted using color: *HP*1F* (pink) and *HP*1S* (teal). These are differentiated by two missense mutations that lead to an isomeric exchange in amino acids (129NE130>129DK130).


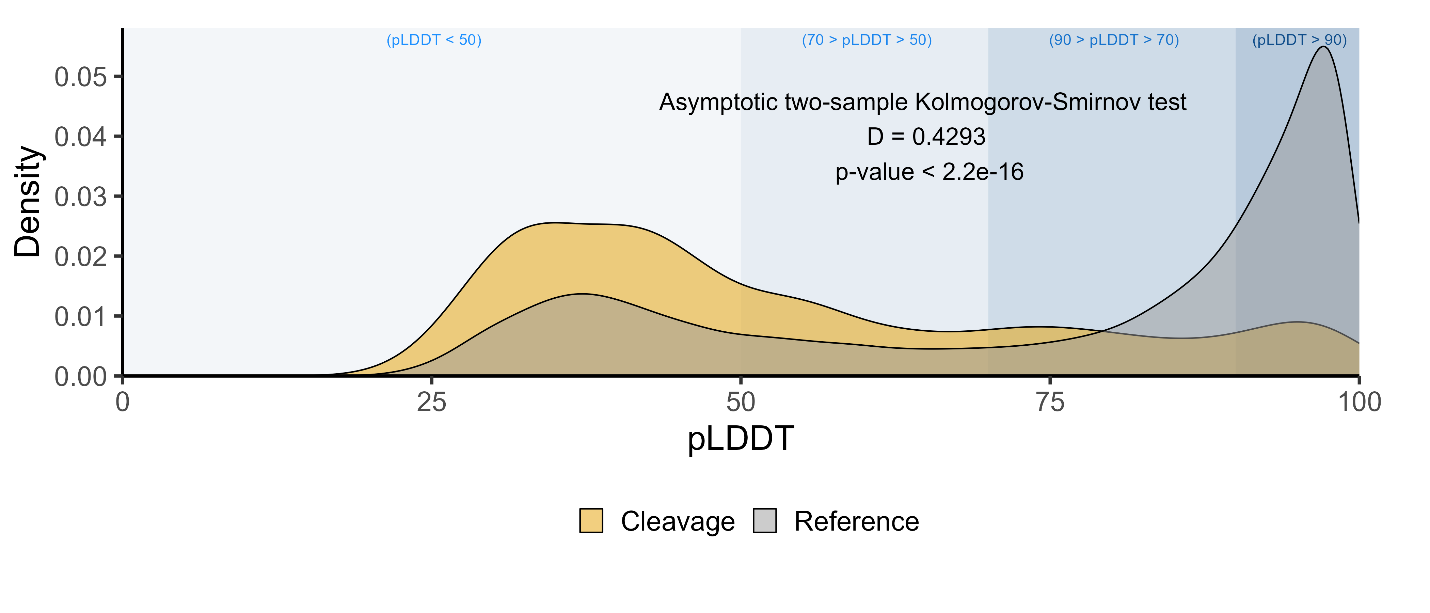


**Supplementary Figure S6.** Density plots for averaged predicted local distance difference test (pLDDT) scores (±3 amino acids surrounding cleavage site) as determined by AlphaFold. Fill color denotes terminal cleavage sites determined by top-down proteomics (gold) or the reference distribution of all amino acids for the detected cleaved genes (gray). Results of asymptotic two-sample Kolmogorov-Smirnov test are shown in plot. Ranges of pLDDT corresponding to very high (pLDDT > 90), high (90 > pLDDT > 70), low (70 > pLDDT > 50), very low (pLDDT < 50) confidence assignments are denoted by backfill color.


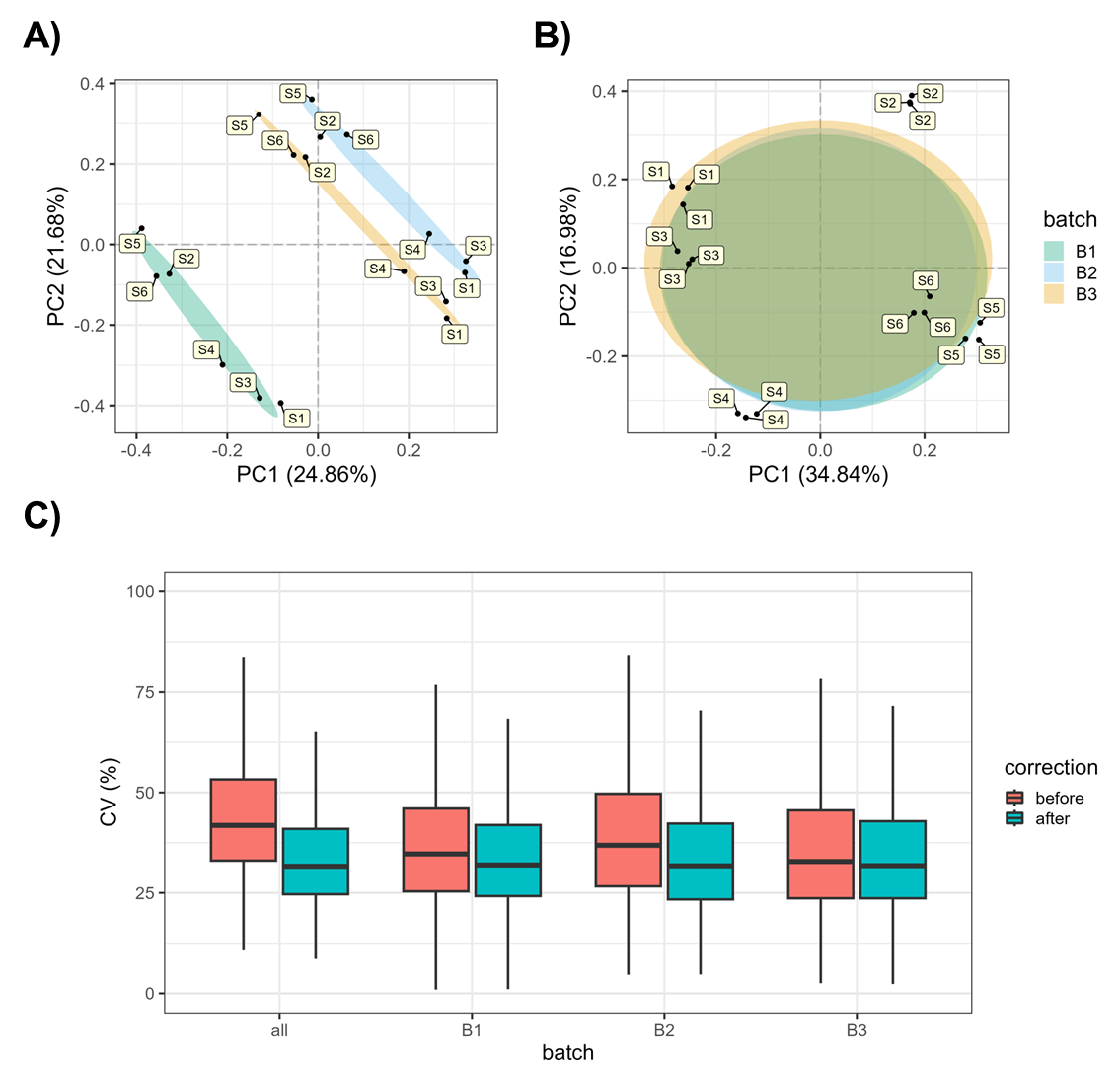


**Supplementary Figure S7.** Assessment of the quantification quality based on 6 mouse brain samples analyzed on LC-MS instrument 3 times separated by 1-3 months in time (batches). PCA plots of relative protein abundances before **A)** and after **B)** batch correction. Samples colored according to the batch. **C)** Variation due to batch effect is effectively eliminated using ComBat procedure as evident from the sample grouping. Distributions of coefficient of variation values computed for the individual proteins at the level of non-log-transformed intensities. Batch effect correction procedure improved the reproducibility from 42.3 to 31.7% down to within-batch variation.


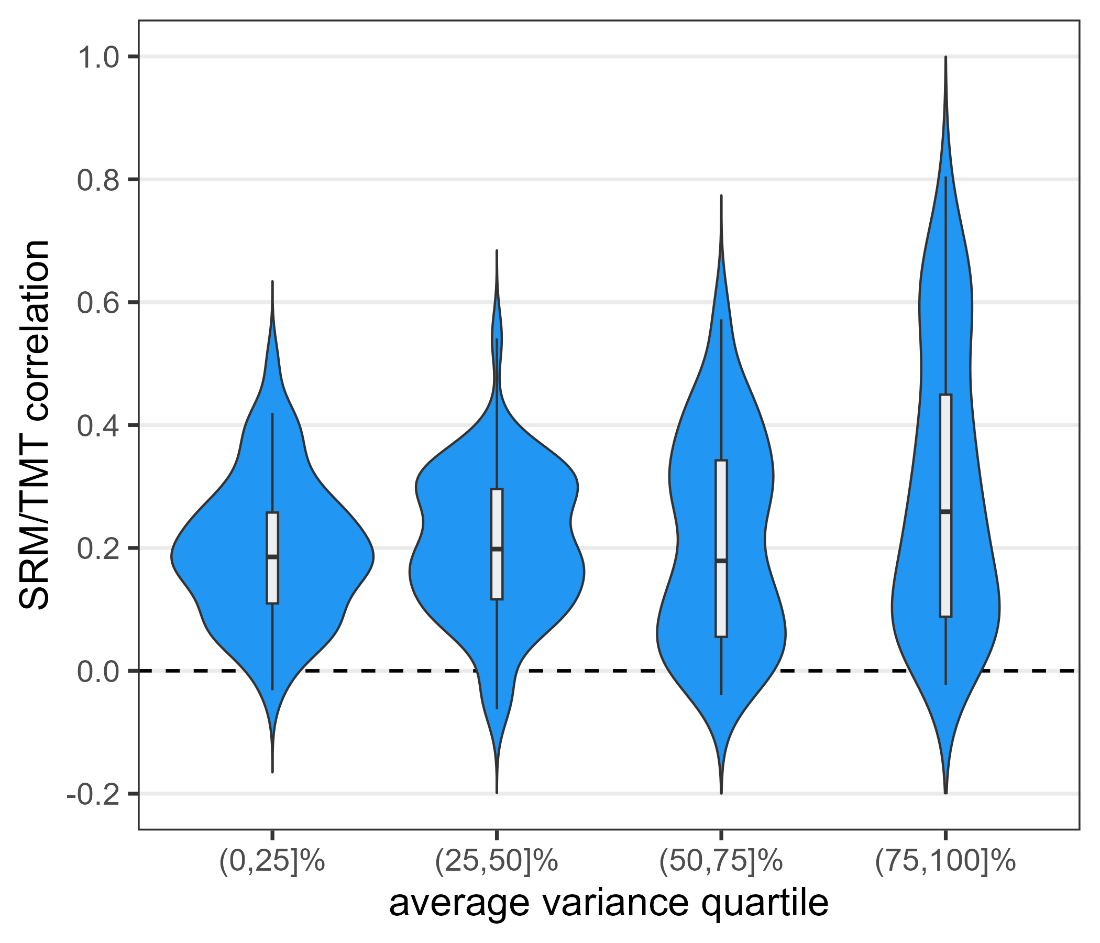


**Supplementary Figure S8.** Densities of the correlations (y-axis) between the SRM and TMT measurements of the 219 proteins across the frontal gyrus of the 400 subjects. The x-axis represents binning of the proteins according to the quartile of the variance of the protein abundances averaged across the SRM and TMT studies. Thus, the proteins that do not change much from sample to sample are in the leftmost bin, while the proteins that strongly change in abundance from sample to sample are in the rightmost bin.


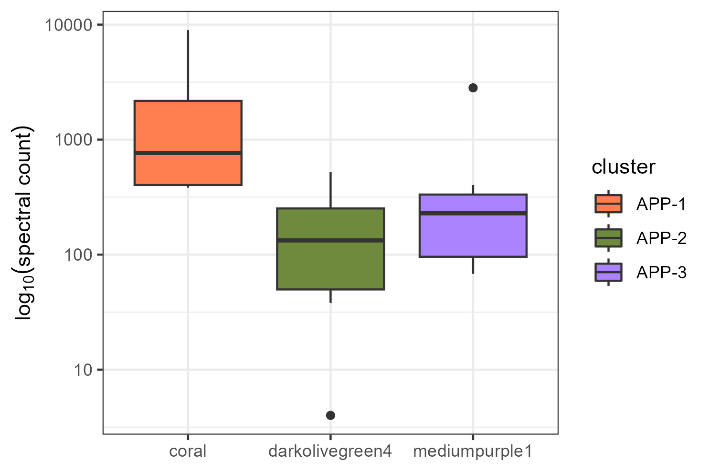


**A)**

**B)**


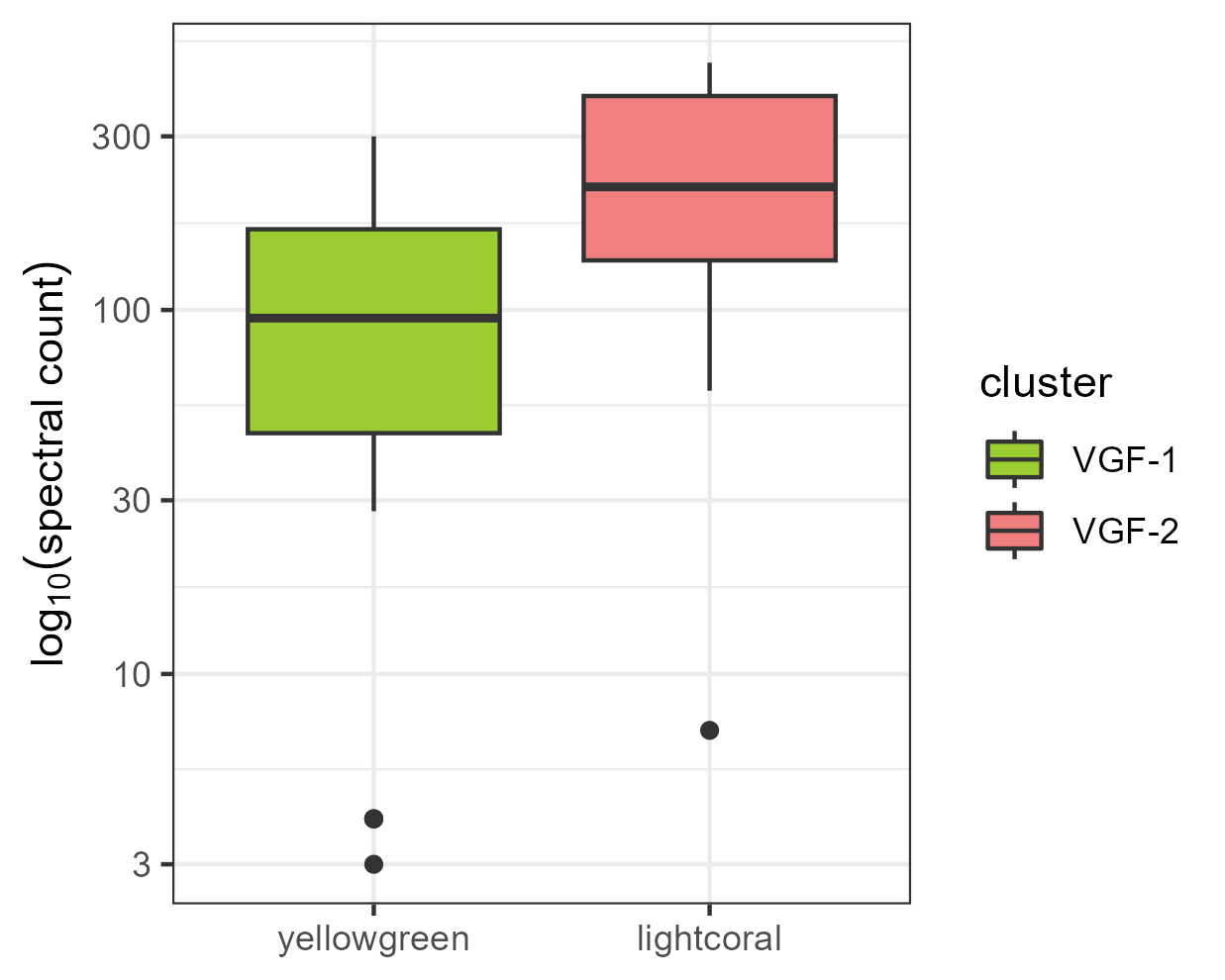


**Supplementary Figure S9.** Estimates on the abundances of the **A)** Aβ and **B)** VGF clusters using spectral counts.

# Supplementary Tables

| Gene (Accession) | Glycan Composition | Mass (Da) |
| --- | --- | --- |
| HBA1 (P69905) | HexN | 161.06 |
| HBA1 (P69905) | Hex | 162.05 |
| HBB (P68871) | Hex | 162.05 |
| HSPE1 (P61604) | Hex | 162.15 |
| TSC22D1 (Q15714) | HexNAc | 203.07 |
| NDUFAB1 (O14561) | Hex(1)HexA(1) | 338.08 |
| CHGA (P10645) | Hex(1)HexNAc(1) | 365.13 |
| HSPE1 (P61604) | Hex(1)HexNAc(1)NeuGc(2) | 980.62 |
| MAP2 (P11137) | dHex(1)Hex(1)HexNAc(2)NeuAc(1)Sulf(1) | 1085.92 |
| EEF1A1 (P68104) | dHex(2)HexHexNAc(2)Kdn | 1110.64 |
| NDUFAB1 (O14561) | *Hex(2)HexNAc(4)Sulf(1) | 1236.51 |
| UBB (P0CG47) | Hex(6)HexNAc(4)Me(3) | 1826.83 |
| TUBA1B (P68363) | Ambiguous | 2311.33 |
| TSC22D3 (Q99576) | Ambiguous | 2783.67 |
| NAP1L1 (P55209) | Ambiguous | 3936.96 |

**Supplementary Table S3**. Proteins with assigned glycan modifications or unknown large modifications that exhibit glycan-like behavior by collision-induced dissociation (CID). Proteins are listed as gene (accession) and glycan interim names from Unimod are listed when available. Monoisotopic masses for detected modifications are listed in daltons (Da). Glycan compositions marked by asterisks (*) are tentative assignments from the GlyToucan dataset based on monoisotopic mass. Abbreviations for glycan composition are as follows: Hex (hexose), HexNAc (N‐acetylhexosamine), HexA (hexuronic acid), dHex (deoxyhexose), NeuGc (N-glycoyl neuraminic acid), NeuAc (N-acetyl neuraminic acid), Kdn (2-Keto-3-deoxy-nononic acid), HexN (hexosamine), Pent (pentose), Sulf (sulfate), Me (methyl).

# References

47 Kandi, S. et al. Amyloid beta Proteoforms Elucidated by Quantitative LC/MS in the 5xFAD MouseModel of Alzheimer's Disease. J Proteome Res 22, 3475-3488, doi:10.1021/acs.jproteome.3c00353 (2023).
